# Ex vivo culture of intact human patient derived pancreatic tumour tissue

**DOI:** 10.1038/s41598-021-81299-0

**Published:** 2021-01-21

**Authors:** John Kokkinos, George Sharbeen, Koroush S. Haghighi, Rosa Mistica C. Ignacio, Chantal Kopecky, Estrella Gonzales-Aloy, Janet Youkhana, Paul Timpson, Brooke A. Pereira, Shona Ritchie, Elvis Pandzic, Cyrille Boyer, Thomas P. Davis, Lisa M. Butler, David Goldstein, Joshua A. McCarroll, Phoebe A. Phillips

**Affiliations:** 1 Pancreatic Cancer Translational Research Group, School of Medical Sciences, Lowy Cancer Research Centre, UNSW Sydney, Sydney, NSW Australia; 2 Australian Centre for Nanomedicine, ARC Centre of Excellence in Convergent Bio-Nano Science and Technology, UNSW Sydney, Sydney, NSW Australia; 3 Prince of Wales Hospital, Prince of Wales Clinical School, UNSW Sydney, Sydney, NSW Australia; 4 Cancer Theme, The Kinghorn Cancer Centre, Garvan Institute of Medical Research, Sydney, NSW Australia; 5 St. Vincent’s Clinical School, Faculty of Medicine, UNSW Sydney, Sydney, NSW Australia; 6 Biomedical Imaging Facility, Mark Wainwright Analytical Centre, Lowy Cancer Research Centre, UNSW Sydney, Sydney, NSW Australia; 7 Australian Centre for Nanomedicine, UNSW Sydney, Sydney, NSW Australia; 8 Centre for Advanced Macromolecular Design, School of Chemical Engineering, UNSW Sydney, Sydney, NSW Australia; 9 ARC Centre of Excellence in Convergent Bio-Nano Science and Technology and Australian Institute for Bioengineering and Nanotechnology, The University of Queensland, Brisbane, QLD Australia; 10 ARC Centre of Excellence in Convergent Bio-Nano Science and Technology, Monash Institute of Pharmaceutical Sciences, Monash University, Melbourne, VIC Australia; 11 Adelaide Medical School and Freemasons Foundation Centre for Men’s Health, University of Adelaide, Adelaide, SA Australia; 12 South Australian Health and Medical Research Institute, Adelaide, SA Australia; 13 Children’s Cancer Institute, Lowy Cancer Research Centre, UNSW Sydney, Sydney, NSW Australia; 14 School of Women’s and Children’s Health, UNSW Sydney, Sydney, NSW Australia

**Keywords:** Pancreatic cancer, Cancer models, Cancer microenvironment

## Abstract

The poor prognosis of pancreatic ductal adenocarcinoma (PDAC) is attributed to the highly fibrotic stroma and complex multi-cellular microenvironment that is difficult to fully recapitulate in pre-clinical models. To fast-track translation of therapies and to inform personalised medicine, we aimed to develop a whole-tissue ex vivo explant model that maintains viability, 3D multicellular architecture, and microenvironmental cues of human pancreatic tumours. Patient-derived surgically-resected PDAC tissue was cut into 1–2 mm explants and cultured on gelatin sponges for 12 days. Immunohistochemistry revealed that human PDAC explants were viable for 12 days and maintained their original tumour, stromal and extracellular matrix architecture. As proof-of-principle, human PDAC explants were treated with Abraxane and we observed different levels of response between patients. PDAC explants were also transfected with polymeric nanoparticles + Cy5-siRNA and we observed abundant cytoplasmic distribution of Cy5-siRNA throughout the PDAC explants. Overall, our novel model retains the 3D architecture of human PDAC and has advantages over standard organoids: presence of functional multi-cellular stroma and fibrosis, and no tissue manipulation, digestion, or artificial propagation of organoids. This provides unprecedented opportunity to study PDAC biology including tumour-stromal interactions and rapidly assess therapeutic response to drive personalised treatment.

## Introduction

Patients with pancreatic ductal adenocarcinoma (PDAC) have less than 9% chance of survival 5 years post-diagnosis^1^. Despite aggressive treatment regimes, there has been little improvement in patient survival in the past 3 decades^2,3^. A critical driver of PDAC tumour aggressiveness and a key barrier to drug delivery is the highly fibrotic stroma which can make up the majority of the tumour mass^4,5^ and is produced by cancer-associated fibroblasts (CAFs)^6^. A PDAC-CAF cell cross-talk network is known to promote the progression, chemoresistance and metastasis of pancreatic tumours^6–8^. This is also a key limitation to pre-clinical evaluation of therapeutics for PDAC, as a majority of pre-clinical in vitro*/*ex vivo PDAC models lack the presence of CAFs and an abundant and functional fibrotic stroma. Thus, in order to fast-track the clinical translation of new drug candidates and to identify existing drugs that will be effective on a patient’s individual tumour, there is an unmet need to develop better pre-clinical models that are: (1) easy to establish; (2) cost-effective; (3) provide results in a timely manner to inform patient treatment; (4) avoid mechanical or enzymatic digestion of tissue; and (5) closely reflect the biology of human disease.

Currently, human-PDAC xenograft mouse models and genetically engineered mouse models (GEMMs) are the gold-standard for pre-clinical drug testing and are highly valuable tools to study PDAC biology^9,10^. Patient-derived xenograft mouse models can reflect inter-patient heterogeneity, but are expensive, time consuming, lack the presence of a functional immune system, and have the extra complexity of infiltrating mouse stroma into a tumour of human origin^11,12^. GEMMs contain a complex multicellular fibrotic microenvironment, however the species genome is not identical to the human genome and they are expensive and time-consuming^9^. Recent years have seen the development of patient-derived organoid models as a pre-clinical model, but these involve the manipulation and mechanical or enzymatic digestion of human tumour tissue and are often derived from a single cell type (tumour cells)^13,14^ which does not fully mimic human PDAC tumours. Most importantly, organoids often lack a fibrotic stroma and the presence of CAFs and blood vessels. Taken together, these limitations highlight the need to develop more clinically relevant models of PDAC to complement the other models available.

Recent evidence highlights that the in situ spatial interaction between tumour and stromal cells in PDAC can provide clinically-important information^15^. Thus, an ideal model should reflect the complex microenvironment and contain all cell types within the same 3D architecture as they were present in a patient’s tumour. Here, we have developed a new PDAC pre-clinical model that retains the 3D architecture of human patient derived PDAC tumours. Importantly, this model does not involve any chemical, enzymatic, or mechanical digestion of PDAC tissue and thus avoids artificially skewing cell populations. We further demonstrate that this model can be applied to test both clinically approved chemotherapy drugs as well as novel therapeutics including a nano-based gene silencing drug developed in our lab. This new model provides a unique opportunity to closely study the biology of the PDAC tumour microenvironment, to identify novel gene targets and test new treatment strategies in a cost-effective and timely manner.

## Results

### Culture and characterisation of human patient derived PDAC explants

**Figure 1:**
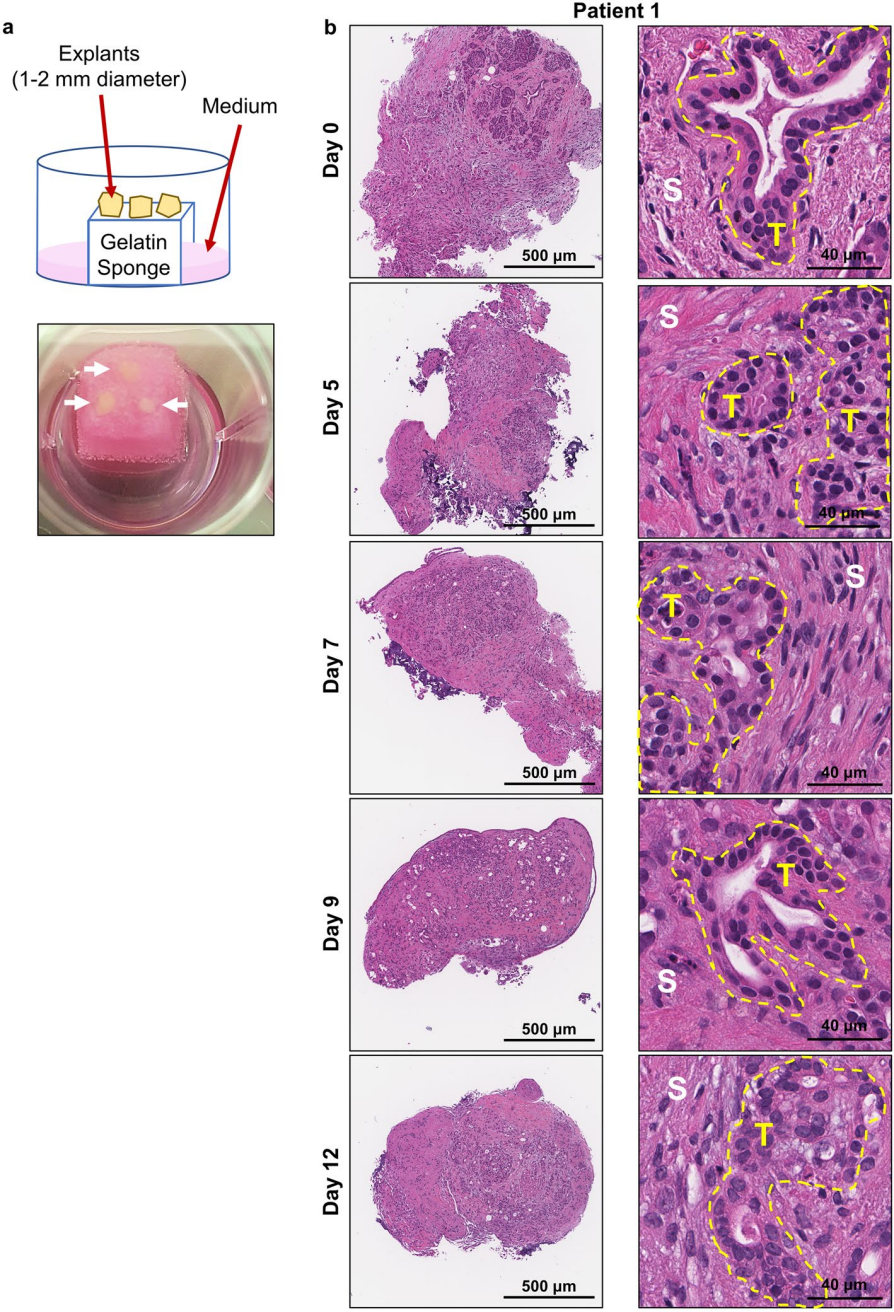
Tumour and stromal architecture are maintained in human patient derived pancreatic ductal adenocarcinoma explants for 12 days of culture. **(a)** Schematic showing the set-up of the ex vivo culture method and a representative photo of a gelatin sponge containing three PDAC explants in a well of a 24-well plate. White arrows point to the three explants on the gelatin sponge during culture. **(b)** Representative H&E images of patient 1 explants at low and high magnification from days 0–12. Tumour elements are outlined in yellow and compartments labelled as tumour (T) and stroma (S).

**Figure 2:**
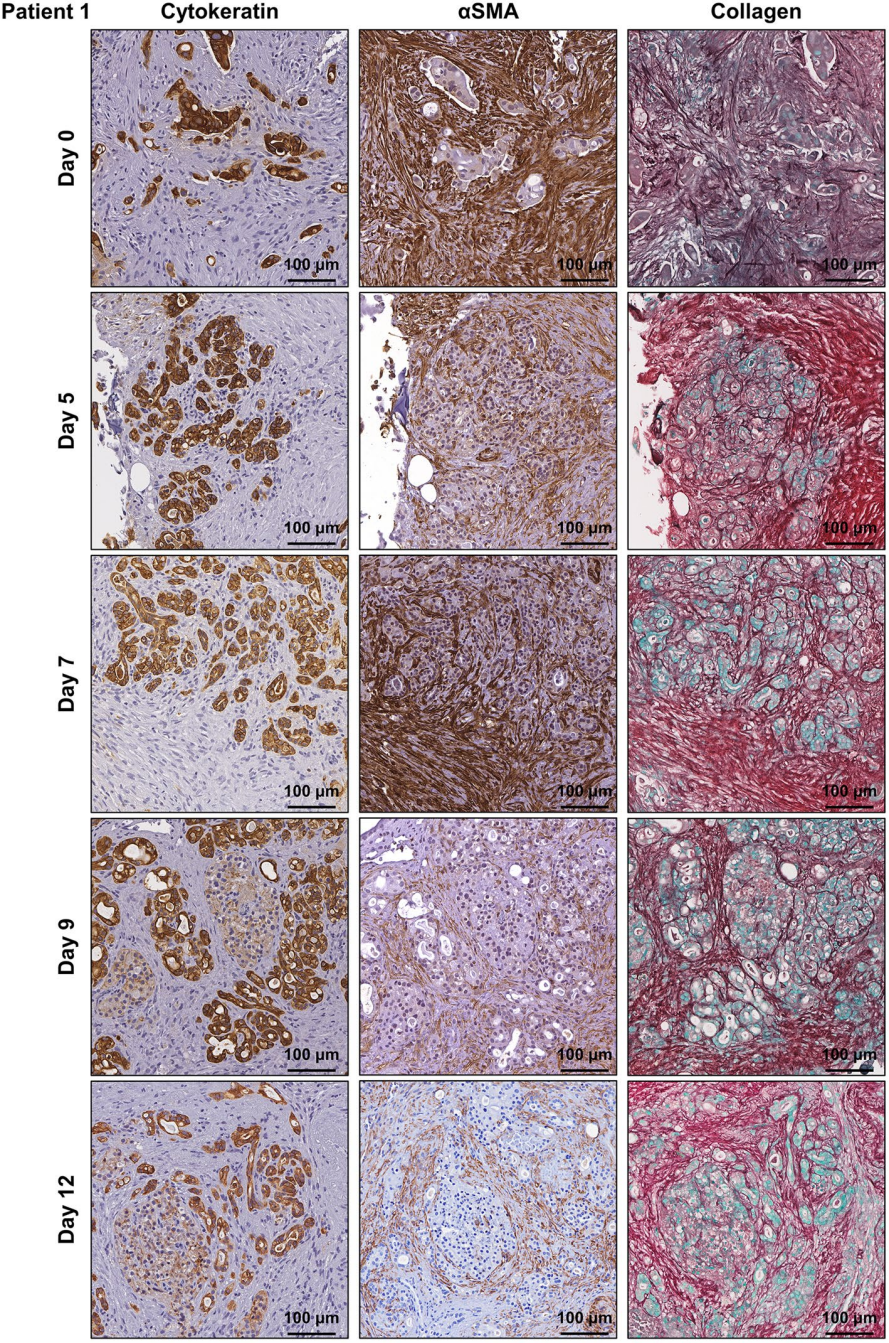
Characterisation of human patient derived pancreatic ductal adenocarcinoma explants cultured for 12 days. Immunohistochemistry was performed for cytokeratin, α-smooth muscle actin (αSMA), and collagen (picrosirius red/methyl green) on patient 1 explants from days 0–12.

**Figure 3:**
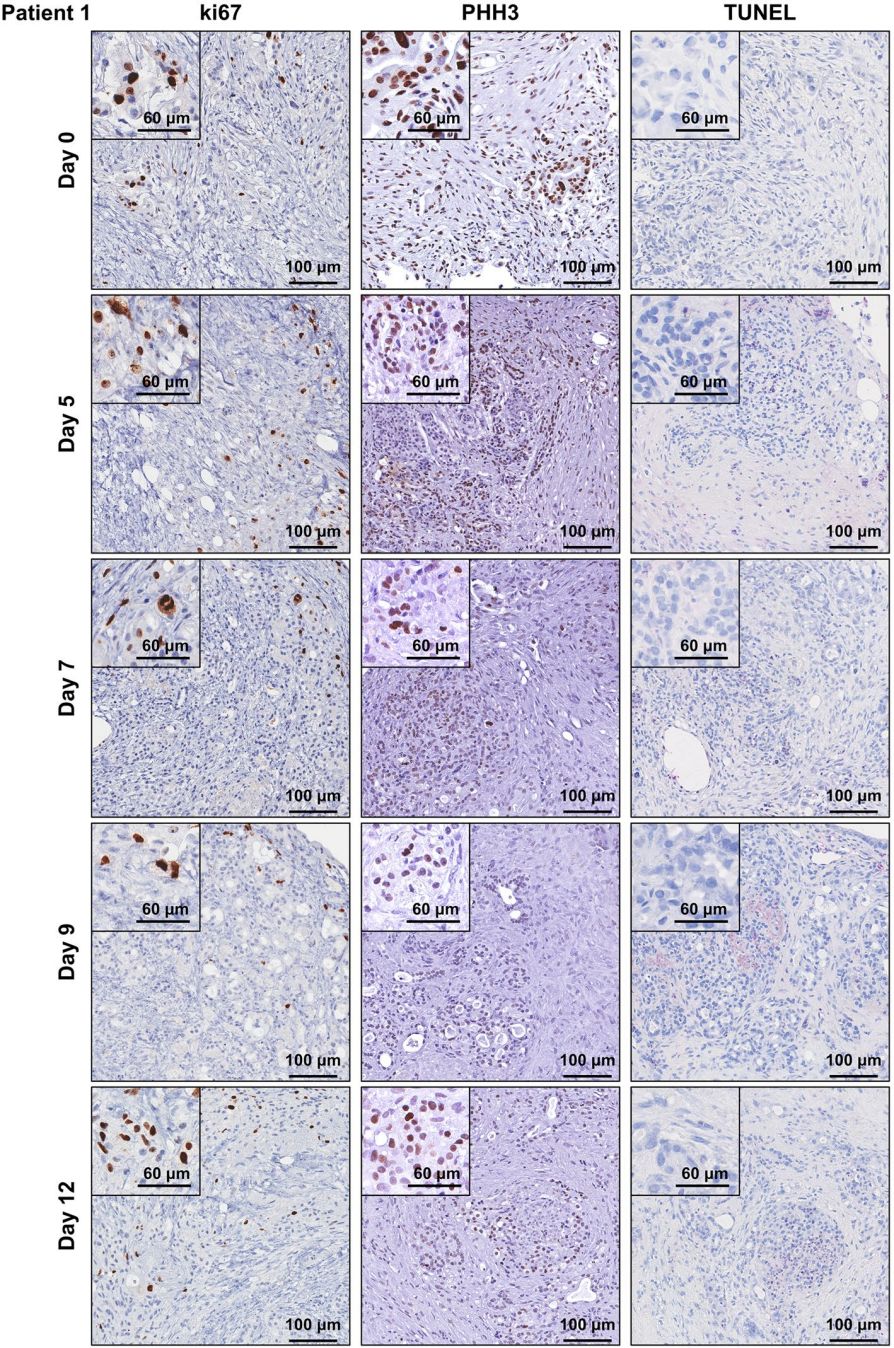
Human patient derived pancreatic ductal adenocarcinoma tumour explants remain viable for 12 days of culture. Immunohistochemistry was performed for ki67, phospho-histone H3 (PHH3) and TUNEL on patient 1 explants from days 0–12. Insets show representative higher magnification views.

**Figure 4:**
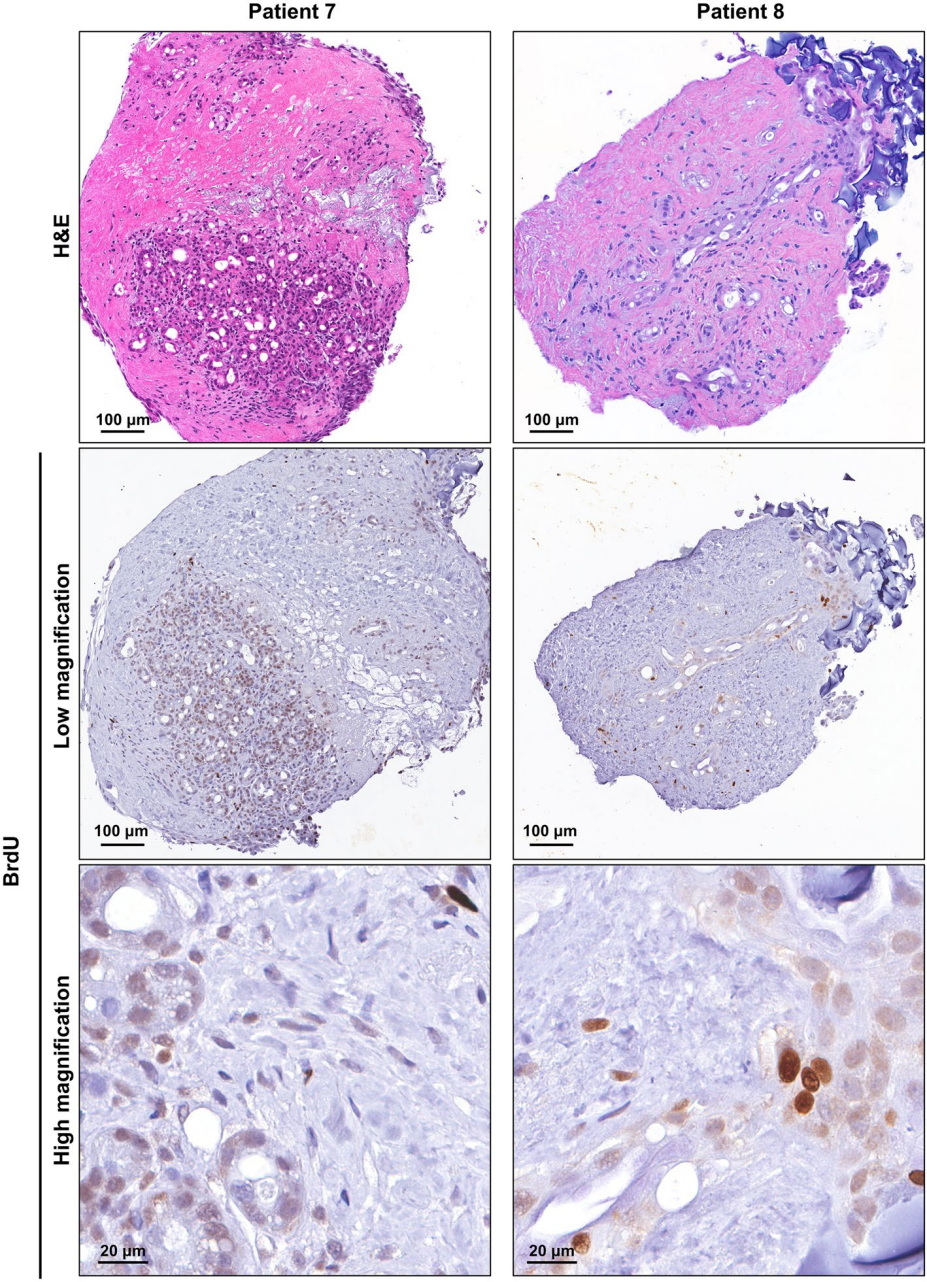
Patient derived pancreatic ductal adenocarcinoma tumour explants demonstrate positive bromodeoxyuridine (BrdU) staining after 12 days of culture. Patient 7 and 8 tumour explants were treated with 10 μM BrdU for 24 h prior to fixation at day 12. Immunohistochemistry demonstrated positive BrdU staining in both tumour and stromal cells in the day 12 explants.

Human PDAC tumour tissue was obtained from patients undergoing surgical resection (pancreaticoduodenectomy) of pancreatic cancer. Patient characteristics are listed in Supplementary Table S1 online. A small piece of tissue was resected from the tumour mass and transported within 15 min to the laboratory on ice. The tumour tissue was cut into 1–2 mm explants and cultured on pre-soaked gelatin sponges for 12 days (Fig. 1a and Supplementary Fig. S1 online). Hematoxylin and eosin (H&E) staining revealed that both tumour and stromal architecture of the patient PDAC explants was retained throughout the 12-day culture (Fig. 1b and Supplementary Figs. S2-S3 online). With similar architecture to the uncultured day 0 controls, we observed in cultured PDAC explants an arrangement of both cytokeratin-positive tumour cells and stromal αSMA-positive CAFs within an abundant distribution of fibrillar collagen (Fig. 2 and Supplementary Figs. S4-S8 online). Importantly, we demonstrated positive staining for two independent cell proliferation markers [ki67 and phospho-histone H3 (PHH3)], and negative staining for a cell-death marker (TUNEL, Fig. 3 and Supplementary Figs. S4-S8 online). A PDAC explant section treated with DNAse served as a positive control for TUNEL (Supplementary Fig. S9 online). To assess the de novo proliferation status of PDAC explants, we treated explants from two PDAC patients with bromodeoxyuridine (BrdU) for 24 h prior to fixation at day 12. We observed positive nuclear staining of BrdU in a majority of tumour cells and a subset of stromal cells at day 12 (Fig. 4).

**Figure 5:**
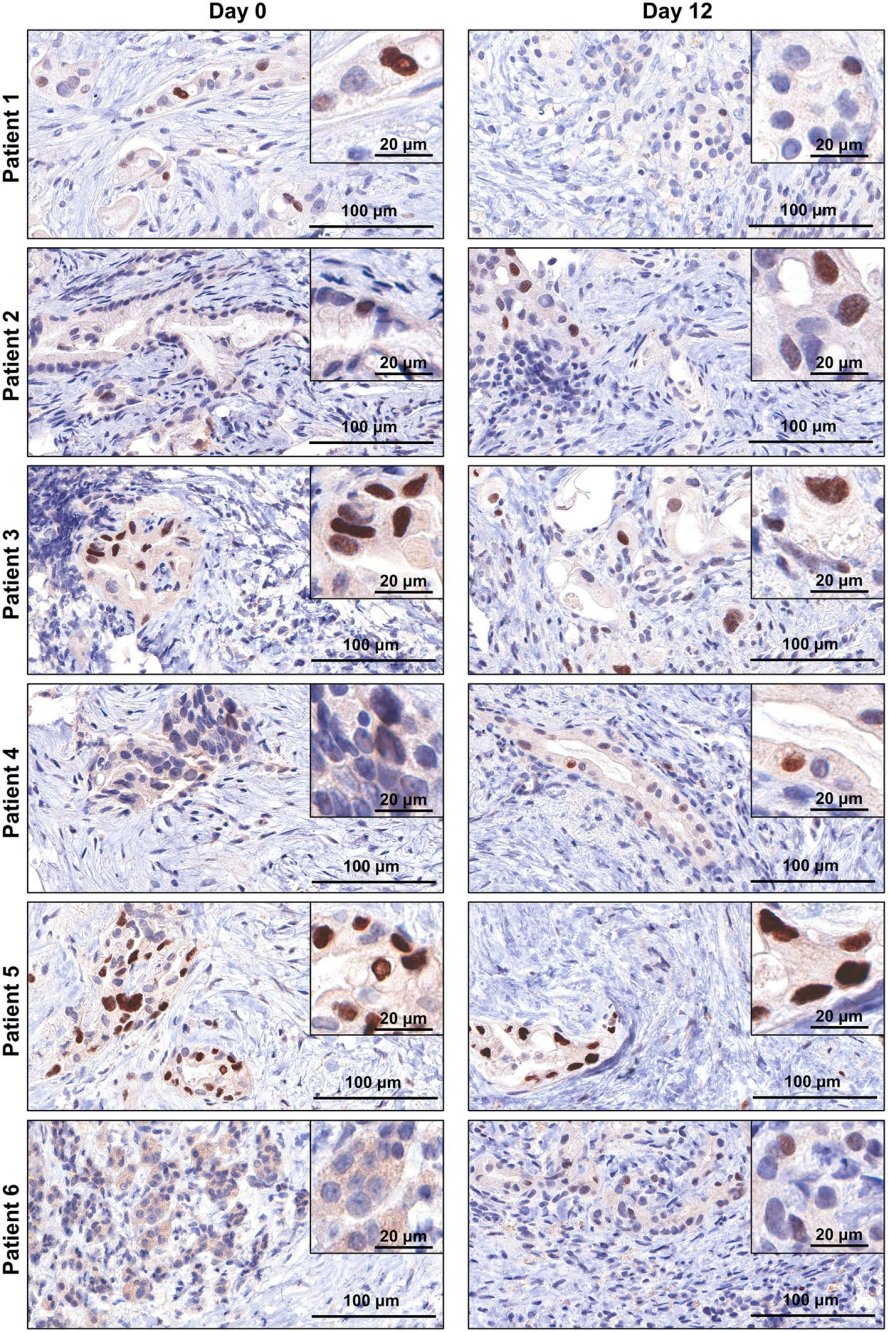
Human patient derived pancreatic ductal adenocarcinoma tumour explants maintain p53 protein status after 12 days of culture. Immunohistochemistry was performed for p53 on day 0 and day 12 tumour explants from patients 1–6. Insets show representative higher magnification views.

We next investigated whether tumour specific drivers are maintained in cultured explants. We performed immunohistochemistry to assess p53 protein status in day 0 and day 12 PDAC explants from patients 1–6. All patient tumour explants contained positive p53 protein in tumour cells at day 0 and after 12 days of culture (Fig. 5).

**Figure 6:**
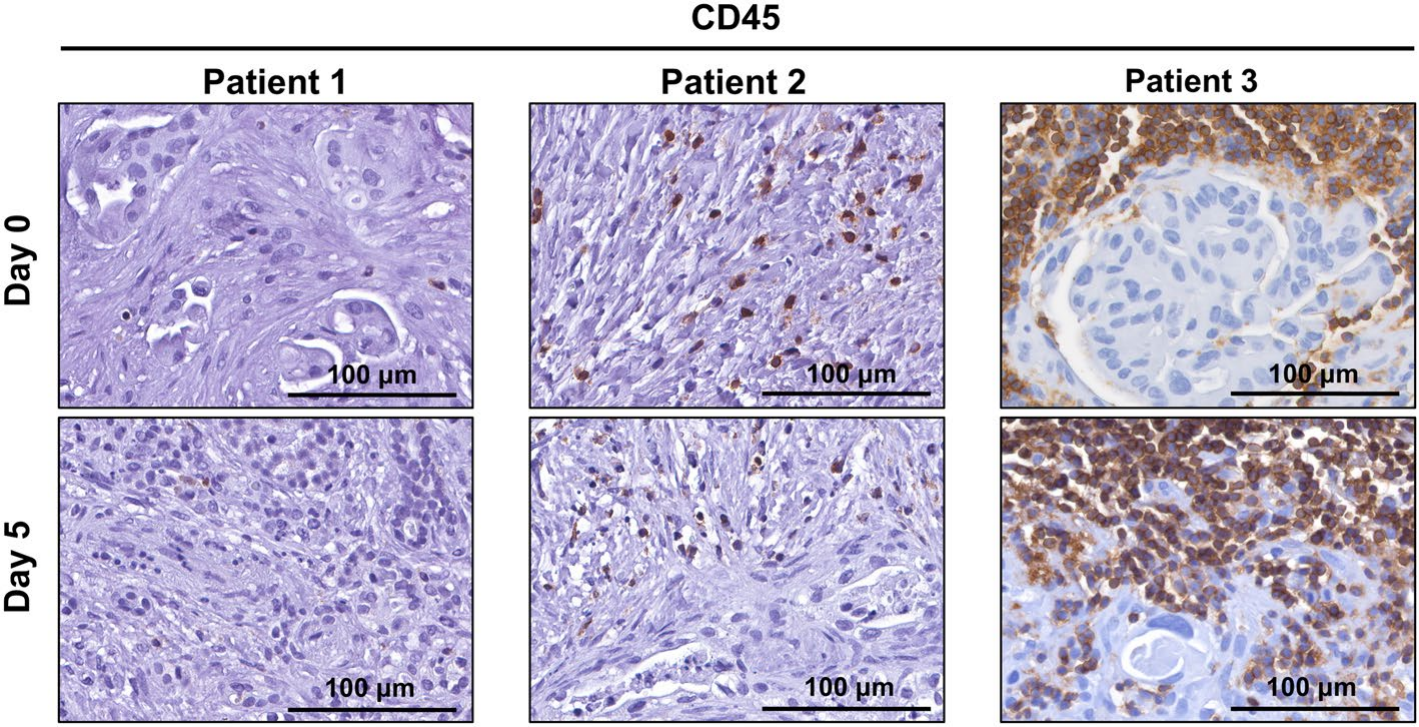
CD45-positive lymphocytes remain viable for 5 days in human patient derived pancreatic ductal adenocarcinoma tumour explants. Immunohistochemistry was performed for lymphocyte marker CD45 on tumour explants from patients 1–3 at day 0 and day 5.

We also performed immunohistochemistry for CD45 (lymphocyte marker) and observed a high degree of lymphocyte infiltrate in the day 0 control explants of patient 3, and this was maintained in explants cultured for 5 days (Fig. 6). Interestingly, according to the surgical pathology report of the resected tumour, patient 3 had a rare loss of MSH6 expression which is known to increase neoantigen presentation and could explain the unusually high amount of CD45-positive immune cells in the tumour. In contrast, patient 1 and 2 explants had significantly less CD45-positive lymphocytes cells. Notably, the amount of immune infiltrate across all patients was maintained between day 0 and day 5 explants. Explants cultured for 7–12 days had very few CD45-positive cells (data not shown), suggesting that the lymphocytes present in the patient’s tumours only remain present for 5 days in our ex vivo explant model.

We also performed light sheet microscopy on day 0 explants from two PDAC patients, demonstrating the ability to image a whole tissue explant in 3D. The 3D reconstruction of the day 0 control tissue explant showed individual cell nuclei with distinct areas of F-actin (phalloidin) expression throughout the explant (Supplementary Fig. S10 online). This could suggest a dispersed arrangement of F-actin rich stromal cells^16^ throughout the tissue. We next proceeded to stain a day 0 explant from another PDAC patient with more specific cell markers (cytokeratin and αSMA). As expected, the 3D reconstruction of the whole tissue explant demonstrated distinct areas of cytokeratin-positive tumour elements and αSMA-positive CAFs (Supplementary Fig. S10 online).

In addition to patients with PDAC, we also cultured explants from three patients with pancreatic neuroendocrine tumours (PNET). H&E staining demonstrated that the architecture of these explants was also maintained after 12 days of culture (Supplementary Fig. S11 online). We stained the day 0 and day 12 PNET explants with a neuroendocrine specific marker (synaptophysin) and although the number of neuroendocrine cells varied between the 3 patients, this was maintained between day 0 and day 12 explants from the same patients (Supplementary Fig. S11 online). We also cultured tumour explants from a patient with a rare metastasis of a leiomyosarcoma to the pancreas, and again H&E staining demonstrated preserved architecture after 12 days of culture (Supplementary Fig. S12 online). Overall, these findings show that the ex vivo explant model can maintain the viability, cell composition, and extracellular matrix of tissue explants from a range of human pancreatic tumours including PDAC and pancreatic neuroendocrine tumours for at least 12 days of culture.

### Testing of clinical and novel therapeutics in pancreatic tumour tissue explants

**Figure 7:**
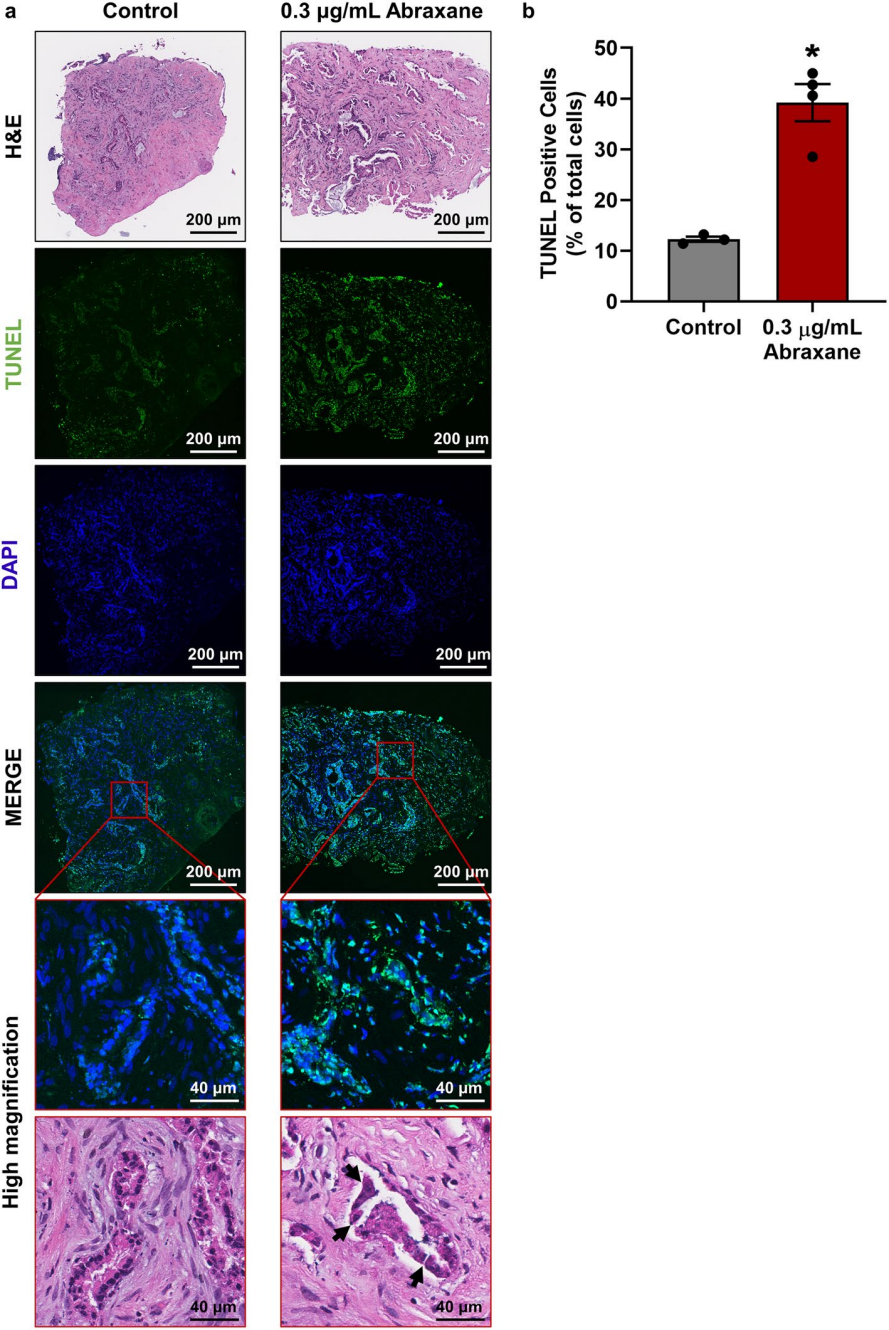
Testing of clinical therapeutics in human patient derived pancreatic ductal adenocarcinoma explants. **(a)** Tumour explants from patient 2 were treated with or without 0.3 μg/mL Abraxane on days 0, 3, 6 and 9, then fixed on day 12. TUNEL staining was performed to assess levels of cell death. Black arrows in H&E stained sections point to areas of ductal fragmentation in Abraxane treated explants. **(b)** Quantification of TUNEL positive cells using QuPath demonstrated increased cell death in Abraxane treated explants compared to untreated controls. A paired t-test was performed to compare TUNEL-positivity in control vs Abraxane treated explants from a single patient, with 3–4 explants per treatment from distinct regions of the patient’s tumour (represented by each dot in the bar graph). Bars represent mean ± S.E.M., *p = 0.0306.

We next investigated whether the ex vivo explant culture model can be used to test clinically approved or novel therapeutics. We first tested Abraxane (human albumin-bound paclitaxel) which is currently one of the chemotherapeutic agents used in first-line therapy for PDAC^17^. Abraxane (0.3 μg/mL or 4.2 μg/mL) was added to the medium reservoir every 3 days and explants were fixed on day 12. We then performed TUNEL staining to assess cell death. PDAC explants from 2 patients (patients 2 and 7) were sensitive to Abraxane with high levels of cell death following Abraxane treatment (39–63% TUNEL positivity, Fig. 7a,b and Supplementary Fig. S13 online). H&E staining revealed architectural changes to tumour cells with Abraxane treatment including fragmentation of ductal elements (black arrows, Fig. 7a). In contrast, we identified a PDAC patient (patient 8) whose explants were non-responsive to Abraxane with less than 6% TUNEL positivity observed in the Abraxane treated explants (Supplementary Fig. S14 online). No clinical treatment information was available for patients 2 and 7, as patient 2 elected to not receive adjuvant chemotherapy, and patient 7 had not yet begun adjuvant chemotherapy at the time of submission of the manuscript. However, for patient 8, clinical information showed that this patient had a Whipple resection prior to the onset of this study (September 2017) for a moderately differentiated adenocarcinoma of the head and neck of the pancreas. The patient received Gemcitabine and Abraxane adjuvant chemotherapy, but the tumour recurred as a moderately differentiated PDAC in the tail of the pancreas which was resected (August 2020), and tumour explants established from this resection.

**Figure 8:**
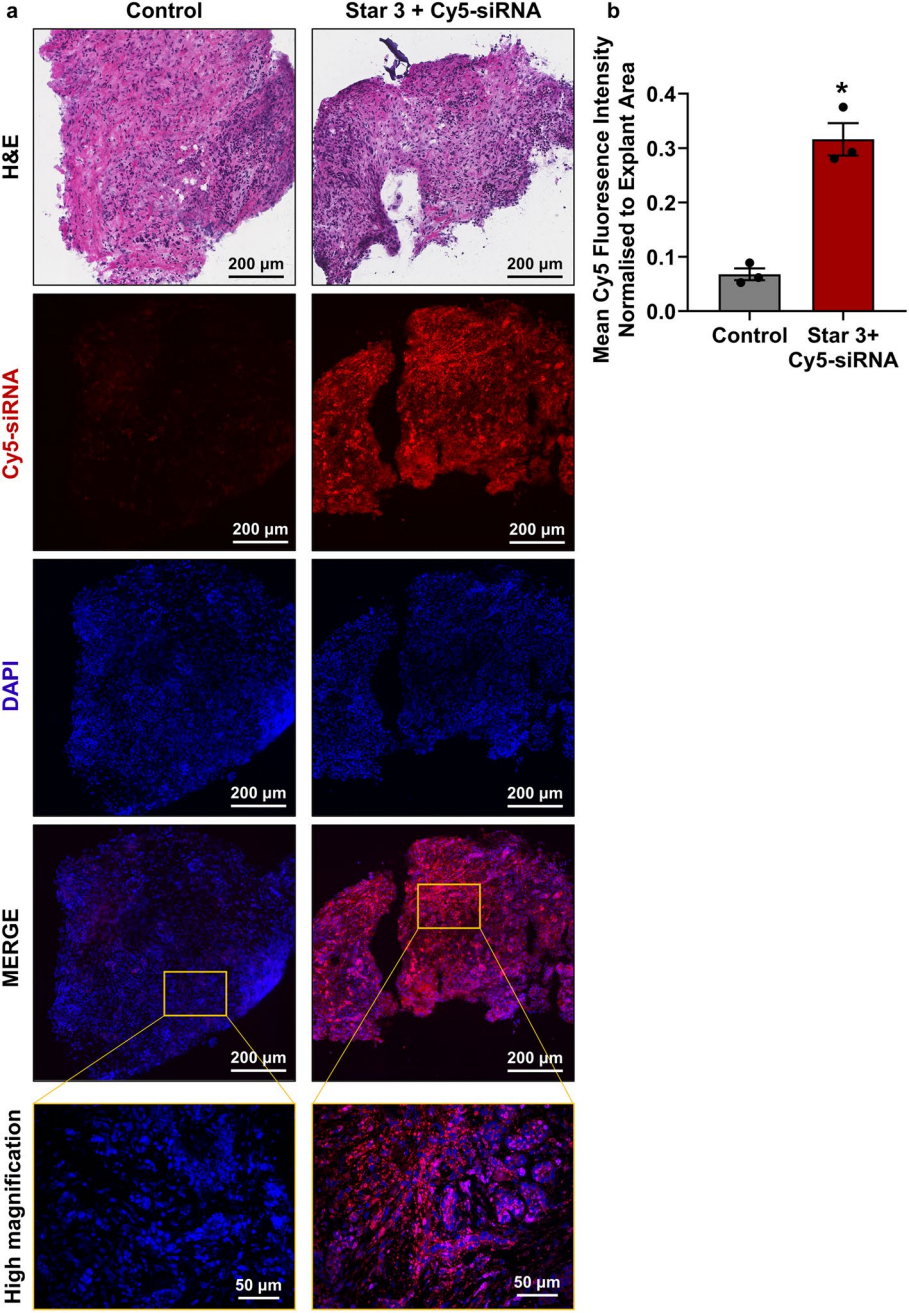
Biodistribution of Star 3 nanoparticles in human patient derived pancreatic ductal adenocarcinoma explants. **(a)** Patient-derived explants from patient 13 were treated for 24 h with or without Star 3 polymeric nanoparticles coupled to Cy5-siRNA. Representative images show uptake of Cy5-siRNA throughout the entire explants. **(b)** Quantification of Cy5 mean fluorescence demonstrated significant uptake of Star 3 + Cy5-siRNA in treated explants compared to untreated controls. A paired t-test was performed to compare mean fluorescence normalised to explant area from a single patient, with 3 explants per treatment from distinct regions of the patient’s tumour (represented by each dot in the bar graph). Bars represent mean ± S.E.M., *p = 0.0176.

Another key potential of our novel ex vivo explant model is the ability to perform real-time mechanistic studies and study cell–cell interactions in an unmanipulated piece of human PDAC tissue. We hypothesised that gene therapeutics such as siRNA would be a useful tool to perform such studies, hence we examined whether our tissue explant model is amenable to transfection with nanoparticle-siRNA^18,19^. A di-block copolymer nanoparticle (Star 3) developed in our lab^18,19^ was complexed to fluorescently labelled siRNA. Human PDAC explants were placed in triplicate on gelatin sponges that had been pre-soaked in medium containing Star 3 complexed to siRNA. After 24 h, tissue explants were processed for frozen tissue sections and Star 3-siRNA uptake assessed by confocal microscopy. As proof-of-principle, we demonstrated that Star 3-siRNA treated tissue explants had abundant uptake of fluorescent siRNA throughout the tissue explants (Fig. 8a and Supplementary Fig. S15 online). Quantification of mean fluorescence from three separate explants from the same patient revealed significantly higher levels of uptake in Star 3 + Cy5-siRNA treated explants compared to untreated controls (4.7-fold increase, p = 0.0176, n = 3 explants per treatment, Fig. 8b).

## Discussion

In this study, we describe the development and characterisation of a novel human pre-clinical model of pancreatic ductal adenocarcinoma (PDAC) that maintains the viability, 3D multicellular architecture and microenvironmental cues of unmanipulated patient derived tumours. This simple and cost-effective model provides unprecedented opportunity to closely study the biology of pancreatic cancer, to identify novel gene therapeutic targets for tumour and stromal cells, to test the anti-cancer activity of novel drugs or combination treatments, and has potential to inform precision medicine for pancreatic cancer. PDAC urgently requires new and more effective treatments. However, for the efficacy of new therapeutic strategies to be evaluated, there is a need for robust pre-clinical models that accurately reflect the biology of the disease in patients. Currently, a broad range of PDAC models are available; ranging from cell lines, GEMMs, patient derived xenografts, and most recently, organoid cultures^9,10,14^. There is no perfect model and each model has its strengths and limitations. For example, 2D in vitro cell line cultures can provide important insights into the signalling pathways dysregulated in cancer cells, but they lack the complexity of a 3D tumour mass. Subcutaneous or orthotopic mouse models of PDAC have the advantage of a 3D arrangement of cancer cells but are often derived from a single cell type. Patient derived xenografts can model interpatient heterogeneity, but are time consuming, expensive, have the complexity of infiltrating mouse stromal cells into a tumour of human origin, and the poor tumour engraftment can potentially bias more aggressive disease^11^. GEMMs have the advantage of spontaneously forming tumours that can mimic patient tumourigenicity in an immune proficient setting but are also time consuming, expensive, and may not always reflect therapeutic response due to mouse and human species differences^9^. Recently, patient-derived organoids have gained much attention for their ability to rapidly model interpatient heterogeneity, but they often consist of tumour cells that have been dissociated from their native extracellular matrix and tumour microenvironment^13,14^. Additionally, most organoids lack the characteristic fibrotic stroma, which is a key drug delivery barrier and is known to drive the progression of PDAC^6–8^. With this in mind, we identified that there is a significant gap in the pre-clinical models available for PDAC, and in particular, there is a need for a model with: (1) abundant and functional stroma, and (2) multicellular architecture with the same 3D organisation as present in human disease.

At the onset of this study, we hypothesised that the ex vivo human patient-derived explant model, established in prostate and breast cancer, has the potential to overcome many of these limitations^20^. This model involves the culture of freshly resected tumour tissue explants (around 1–2 mm diameter) on a gelatin sponge support scaffold soaked in culture media^20^. Nutrients can be taken up by the explants via capillary action through the gelatin sponge, preventing the need to submerge the tissue in culture media which often leads to its degradation within a few days. This model has been used to culture prostate, breast and ovarian cancer explants, but has yet to be translated to PDAC^20–24^.

Recently, a study by Misra et al. (2019)^25^ cultured 350 μm thick PDAC tumour tissue-slices on a cell-culture Millicell insert for up to 96 h. While this was the first study to culture whole-tissue slices of PDAC and represents significant progress in developing more clinically relevant models of PDAC, the culture was only maintained for 96 h and low levels of tissue death were observed from as early as 24 h of culture^25^. A follow up study demonstrated that these cultured PDAC tissue slices have genomic stability with minimal transcriptome changes throughout the 72 h of culture^26^. Several other studies have developed in vitro co-culture models containing tumour cells, cancer-associated fibroblasts (CAFs), and endothelial cells^27–31^, but these models do not perfectly mimic the in situ complexity and heterogeneity of human PDAC tumours. For example, an impressive study by Gupta et al.^27^ developed a co-culture model of cancer cells, endothelial cells and CAFs on a polyurethane scaffold. While such models can be useful for high-throughput screening of therapeutics, they consist of cells artificially dissociated from their native architecture and microenvironment which may not represent the complexity of human disease.

Here, we report for the first time that the gelatin sponge ex vivo explant culture method can be used to culture human patient derived PDAC whole-tissue tumour explants for up to 12 days. We cultured human PDAC explants derived from patient tumours immediately following surgical resection of the tumour. Remarkably, both the tumour and stromal architecture of the explants were retained throughout the 12-day culture, and this was reproduced across six PDAC patients. Indeed, the tissue explants showed dispersed cytokeratin-positive tumour elements surrounded by a dense arrangement of αSMA-positive CAFs and an abundant network of fibrillar collagen, which was highly comparable from days 0–12. Using multiple orthogonal approaches, we comprehensively demonstrated that the proliferative status of both tumour cells and stromal cells is maintained throughout the 12 days culture. We highlighted this by observing positive staining for ki67 and phosphorylated-histone H3 (marker of mitotically active cells^32–34^), as well as bromodeoxyuridine DNA incorporation providing strong evidence that cells display de novo cell proliferation after 12 days of culture.

Immunohistochemistry also revealed that p53 protein status in tumour cells is maintained between day 0 and day 12 explants. Immunohistochemistry has been used to assess p53 status in PDAC and other cancers, as the mutated form of p53 protein is known to accumulate in the nucleus of tumour cells and is detectable by immunohistochemistry, whereas the wild-type protein is rapidly degraded^36–39^. Thus, by demonstrating positive p53 staining in tumour cells between day 0 and day 12 PDAC explants, and consistent with previous findings in ex vivo cultured PDAC slices^26^, our results suggest that tumour specific drivers such as p53 (which is mutated in approximately 75% of PDAC patients^40,41^) are maintained in our PDAC explant model. Future studies will be required to examine the presence of other established PDAC tumour genetic drivers in our explant model.

While PDAC is generally characterised by an immunosuppressive microenvironment with limited intratumoural immune cell populations, we cultured explants from a patient with a rare loss of MSH6 expression (patient 3) where we observed a prominent infiltrate of CD45-positive lymphocytes. Loss of MSH6 results in a deficiency in mismatch repair which can increase neoantigen presentation on tumour cells and promote T-cell infiltration^42^. Notably, this immune population was maintained up to 5 days of culture, thus providing a window to potentially study the effects of immunotherapy on a 3D culture of human PDAC tissue. There is also potential to add patient autologous immune cells to the explant culture and study how they interact with different cell types and the effects of stromal remodelling on immune cell activity and invasion.

Taken together, by highlighting that the overall pathology, viability, 3D multicellular architecture and genetic drivers are maintained in our PDAC tumour explant model, we demonstrate that there is no drift in the phenotype or genotype of the tumour explants over time. This is an advantage over patient derived xenograft models which have been shown to lose human stroma and replace it with mouse stroma^12^. Our results also highlight the low likelihood of dramatic cellular changes occurring within the cultured PDAC explants. While there may be subsets of cells that undergo senescence or cell-cycle changes within the explants, we expect them to be in a similar fraction of cells at baseline in the original patient’s tumour and as known to occur in human PDAC tumours in situ^35^.

Interestingly, we also demonstrated that our model can be used to culture pancreatic neuroendocrine tumour explants as well as tissue explants derived from a patient with metastatic leiomyosarcoma to the pancreas, providing an opportunity to study other pancreatic malignancies which also have poor patient outcome.

The ability to maintain the viability of a patient’s tumour in the lab over a 12-day window is a major advancement and has the potential to revolutionise pancreatic cancer research. Firstly, this model provides an unprecedented opportunity to perform real-time mechanistic studies to better understand interactions of tumour cells with their surrounding stromal and immune cell populations in the same native 3D architecture as they were present in a patient’s tumour. We also propose that the ex vivo tissue explant model can become an integral component of the drug development pipeline as it allows for the potential of new drugs to be evaluated within the context of the major cell types present in an individual patient’s tumour. Inter-patient heterogeneity can be recapitulated in the explant model, allowing the applicability of a new drug to be assessed and to inform which patients would likely benefit from a proposed new treatment. Live tracking of treatment response could also be possible by sampling the medium reservoir and analysing secreted factors related to cell death and senescence as a measure of treatment response.

We investigated whether our tissue explant model is amenable to transfection with nano-based gene silencing drugs. We showed that polymeric nanoparticles which can deliver siRNA to PDAC cells both in vitro and in vivo^18,19^, delivered siRNA throughout the human PDAC tumour explants. No obvious toxicity from the nanoparticle treatment was observed. Thus, the potential to administer therapeutics to patient-derived explants in this model may provide an opportunity to evaluate a novel therapeutic agent before using expensive and time-consuming mouse models. From a nanomedicine perspective, this model holds potential to test the interaction of nanoparticles with all cell types present in a tumour, and to observe their biodistribution in a clinically relevant 3D piece of human tumour tissue. This information is critical, especially in PDAC, to facilitate the translation of nanomedicines to the clinic^43^. In addition, the potential to transfect patient derived human PDAC tumour explants with siRNA or other gene modifying agents (e.g. miRNA mimics/inhibitors, DNA or CRISPR/Cas) provides an opportunity to perform real-time mechanistic studies in tumour tissue containing a 3D multicellular architecture and complex microenvironment.

Most importantly, the PDAC explant model has potential to inform a personalised medicine program for PDAC. Patient-derived xenograft models are being investigated as a tool to guide personalised medicine in other cancer types^44^, but the length of time required to establish these models makes them unsuitable for cancers such as PDAC that have such poor survival. Recent years have seen the focus shift to organoid cultures to inform individual patient treatment^13,45–49^, but a limitation of most organoid models is that they lack the presence of stroma—a major drug delivery barrier for PDAC. While organoids may reflect tumour cell intrinsic resistance to a given chemotherapy agent, a patient’s tumour cells deemed to be “sensitive” to a chemotherapeutic may in fact be resistant in the presence of a fibrotic stroma containing CAFs and immune cells that are well known to cross-talk with tumour cells to promote chemoresistance.

As a proof-of-principle, we demonstrated that patient-derived tissue explants can be treated with Abraxane and anti-cancer activity measured by TUNEL staining as a readout for cell-death. We performed Abraxane treatment in explants from three PDAC patients, two of which showed clear sensitivity to Abraxane whereas the third patient’s explants were non-responsive to Abraxane. Intriguingly, the patient whose explants were non-responsive to Abraxane had recurrence of PDAC following Gemcitabine and Abraxane adjuvant chemotherapy. This suggests that the patient’s tumour was not responsive to Abraxane which was successfully recapitulated in our tumour explant model. Future studies will assess the ability of our explant model to predict patient response to drug treatments in the clinic and to inform personalised therapies for PDAC.

Importantly, this model is both cost effective and time efficient. Preparation of the explants can be completed in approximately 30 min after receiving the surgical sample, so a team of two researchers could process explants from multiple patients within a given week. In our experience, we were able to obtain at least 30 explants from each patient which could allow around 10 different treatment groups to be established from each patient with three explants per treatment group to ensure tumour heterogeneity is reflected.

In addition to informing precision medicine, our PDAC tumour explant model may also provide insight into how chemotherapy treatment affects the microenvironment of PDAC tumours. This has been studied using the same model in human prostate cancer explants and revealed patient-specific changes to the stroma and microenvironment following chemotherapy treatment^50^. Another potential of the PDAC explant model is the opportunity to investigate stromal reprogramming agents by evaluating the effects on CAFs and the extracellular matrix in a model that uniquely maintains the tumour and stromal architecture of patient derived tissue.

While the pancreatic tumour explant model established in this study holds promise for PDAC research, a limitation is that we have only collected tumour samples from patients with surgically resectable disease which represents approximately 15–20% of all PDAC patients and these patients have a better prognosis compared to those with unresectable PDAC^2,41^. Future studies should focus on developing and strengthening collaborations between scientists and clinicians with the aim to obtain fresh tumour tissue from unresectable patients through biopsy of their primary tumour and/or metastatic tumours.

Nonetheless, this study has established a promising new pre-clinical model of PDAC that retains the native 3D multicellular architecture of human pancreatic tumours over 12 days of culture. While we do not believe that this model replaces the need for mouse models, we propose that the ex vivo tissue explant model is a clinically relevant complement to currently available pre-clinical models. Importantly, the ex vivo tissue explant culture method can answer fundamental biological questions and has potential to guide a precision medicine program for PDAC.

## Material and methods

### Ex vivo explant culture

Prior to collection of tumour tissue, haemostatic gelatin sponges (Johnson & Johnson, Cat. JJ-12505) were briefly submerged in culture medium containing high-glucose DMEM, 10% FBS, 5 mM GlutaMAX, 0.01 mg/mL hydrocortisone, 0.01 mg/mL insulin and 1 × antibiotic/antimycotic solution (all from Sigma-Aldrich). Once sponges were soaked, they were placed in 24-well plates and 500 μL culture medium was added to each well to cover the bottom-half of each sponge and stored in a 37 °C/5% CO_2_ incubator until required.

De-identified tumour samples were obtained from patients undergoing a pancreaticoduodenectomy (Whipple procedure) at Prince of Wales Hospital or Prince of Wales Private Hospital, Randwick, NSW, Australia. Patients provided informed consent through the Health Science Alliance Biobank, all work was approved by UNSW human ethics (HC180973) and all experiments were performed in accordance with the relevant guidelines and regulations. Tumour tissue was immediately placed in ice-cold PBS containing 1 × antibiotic/antimycotic solution and transported on ice. Regions of normal pancreas or fat tissue were dissected away from the tumour sample. The remaining tumour tissue was cut into 3 pieces of equal size. The 3 pieces were placed in separate petri dishes labelled as “L”, “M”, or “R”. Each of these 3 pieces were then cut using a scalpel into explants with diameter ranging from 1–2 mm. Explants from the “L” piece were all placed on the bottom left corner of each pre-soaked gelatin sponge, explants from the “M” piece placed in the middle region, and explants from the “R” piece placed in the bottom right corner as described in Supplementary Fig. S1 online. This ensured that each gelatin sponge contained explants from three distinct regions of the tumour tissue to account for intratumoural heterogeneity. A single explant from each of the “L”, “M”, and “R” pieces was immediately fixed in 4% paraformaldehyde and was designated the day 0 control. Once all explants had been placed on the sponges, the 24-well plate was placed in a 37 °C 5% CO_2_ incubator. Approximately 30–50 explants could be obtained from each patient. Culture medium was replaced daily, with fresh insulin, hydrocortisone and antibiotic/antimycotic solution added to the medium immediately prior to it being added to the culture plate. Cultured explants were fixed in 4% paraformaldehyde on days 5, 7, 9 and 12 post-establishment of the model for patients 1–3 and on day 12 for patients 4–12. Fixed explants were embedded in paraffin and 5 μm slices were prepared at the Histopathology Service at the Garvan Institute of Medical Research. All explants were stained with Haematoxylin and Eosin (H&E) for visualisation of explant architecture.

Patient derived explants were treated with or without Abraxane (Specialised Therapeutics Australia; 0.3 or 4.2 μg/mL) by adding Abraxane to the medium used to pre-soak the gelatin sponges on day 0, and 500 μL medium containing Abraxane was added to each well. Abraxane treatment was repeated on days 3, 6 and 9. The 0.3 μg/mL dose of Abraxane was chosen to match concentrations used in vitro. The 4.2 μg/mL dose of Abraxane was based on the maximum plasma concentration of Abraxane after a 30-min intravenous infusion at a dose of 100 mg/m^2^ in human clinical pharmacokinetic data^51^. To assess BrdU DNA incorporation, explants were treated with 10 μM BrdU substrate (BD Biosciences, Cat. 550891) for 24 h prior to fixation on day 12.

### Immunohistochemistry of patient-derived pancreatic tumour explants

Patient-derived explant tissue sections were stained for cytokeratin (DAKO, Cat. M3515; 1:100 overnight at 4 °C), α-smooth muscle actin (αSMA) (Sigma-Aldrich, Cat. A5228; 1:1000 1-h at room temperature), phospho-histone H3 (PHH3) (Cell Signalling, Cat. 53348; 1:200 overnight at 4 °C), bromodeoxyuridine (BrdU) (DAKO, Cat. M0744; 1:50 dilution overnight at 4 °C), CD45 (ThermoFisher Scientific, Cat. 14-9457-82; 1:100 overnight at 4 °C), or synaptophysin (DAKO, Cat. A0010; 1:100 overnight at 4 °C). Briefly, tissue sections were deparaffinised at 60 °C for 30 min then rehydrated through consecutive washes in xylene, ethanol, and water. Antigen retrieval was performed by microwaving slides for 4 min in 10 mM citrate buffer + 0.05% Tween-20 at pH6.0, followed by a 30-min incubation at 104 °C. Non-specific peroxidase activity was blocked with 1% hydrogen peroxide + 1% methanol for 10 min at room temperature. For PHH3 and BrdU staining, tissue sections were permeabilised in 0.5% triton X-100 (Sigma-Aldrich) for 15 min. An additional DNA hydrolysis step was performed for BrdU staining by incubating sections in 3 U/mL DNAse1 for 1 h at 37 °C prior to blocking. After blocking in 10% goat serum, tissue samples were stained with primary antibodies as indicated above and isotype control antibodies (mouse IgG2A, mouse IgG1A, and rabbit IgG) were used as negative controls (representative images shown in Supplementary Fig. S9 online). Biotinylated anti-rabbit (Vector laboratories, Cat. BA-1000) or anti-mouse (DAKO, Cat. E0433) secondary antibodies (1:100) were followed by incubation with Vectastain ABC kit (Vector laboratories). 3,3′ diaminobenxidine (DAB) was used as the substrate, and tissues were counterstained with hematoxylin. Ki67 (ThermoFisher Scientific, Cat. RM-9106; 1:1000 dilution) and p53 (Merck Millipore, Cat. OP03; 1:100 dilution) staining was performed on a LeicaBond RX Autostainer using a Leica Bond Polymer Refined Detection kit. Antigen retrieval was performed using the Leica Bond Epitope Retrieval ER2 at 100 °C for 30 min. All stained tissue sections were scanned on an AperioXT (Leica Biosystems) or Vectra Polaris (PerkinElmer) slide scanner.

TUNEL staining was performed according to manufacturer’s instructions (Sigma-Aldrich, Cat. 11684809910). Antigen retrieval was performed with 20 μg/mL proteinase K for 15 min at 37 °C. Positive control slides were treated with 3 U/mL DNase1 (New England Biolabs, Cat. M0303) for 10 min at room temperature. Tissue sections were incubated for 1 h at 37 °C with TUNEL enzyme diluted 1:2 in TUNEL dilution buffer. For assessment of TUNEL fluorescence (as performed for Abraxane treatment experiment), tissue sections were mounted with ProLong Gold antifade mounting medium with DAPI (Invitrogen, Cat. P36931) and fluorescence scanned on a Vectra Polaris (PerkinElmer) slide scanner. QuPath software was used to quantify the amount of TUNEL-positivity in the whole-tissue explants as a percentage of DAPI-positive cells. A paired t-test (GraphPad Prism 8) was performed to compare TUNEL-positivity in control vs Abraxane treated explants from a single patient, with 3–4 explants per treatment from distinct regions of the patient’s tumour. For the remainder of tissue sections, TUNEL staining was followed by a 30-min incubation with alkaline phosphatase converter for 30 min at 37 °C. Fast Red (Acam, Cat. ab64254) was used as the substrate, and tissue sections were counterstained with haematoxylin.

### Picrosirius red staining of collagen

Patient-derived explant tissue sections were stained with 0.1% picrosirius red for fibrillar collagen and counterstained with methyl green through the UNSW Mark Wainwright Analytical Centre Biomedical Imaging Facility (UNSW Sydney).

### Uptake of polymeric nanoparticle-siRNA complexes in human patient derived PDAC tumour explants

Polymeric nanoparticles (Star 3) were synthesised as previously described^19^. Star 3 nanoparticles (62.5 μg) were complexed for 5 min with 25 μg Cy5-labelled siRNA (Dharmacon custom Luc2 siRNA; Cy5-GCUUAGGCUGAAUACAAAUUUU). The complexed Star 3 + Cy5-siRNA was added to the medium used to soak the gelatin sponges immediately prior to addition of the explants to the sponges and 500 μL of medium containing Star 3 + Cy5-siRNA was then added to each well. After 24 h, explants were embedded and frozen in Tissue-Tek Optimal Cutting Temperature Compound (OCT; VWR International). OCT-embedded Sects. (10 μm thick) were mounted with ProLong Gold antifade mounting medium with DAPI (Invitrogen, Cat. P36931). Fluorescent siRNA uptake was visualised on a Zeiss 900 confocal microscope by taking at least 3 representative images from each explant. Whole-tissue explants were scanned on a Vectra Polaris (PerkinElmer) slide scanner. Quantification was performed on ImageJ by quantifying the mean fluorescence normalised to explant area of the scanned whole-tissue explants. A paired t-test (GraphPad Prism 8) was performed to compare fluorescence of Star 3 + Cy5-siRNA treated explants with untreated explants from a single patient, with 3 explants per treatment from distinct regions of the patient’s tumour.

### Light-sheet microscopy

Fixed human PDAC explants were embedded in agarose and de-lipidation and optical clearing of the explants was performed as previously described^52^. Optically cleared samples were then stained with AlexaFluor-647-conjugated F-actin antibody (1:200, ThermoFisher) and Hoechst (1:300,ThermoFisher) or Cy3-conjugated αSMA antibody (1:400, Sigma-Aldrich) and AlexaFluor-488-conjugated cytokeratin antibody (1:400, BioLegend) in PBS for 7 days at 37 °C. The samples were then equilibrated in reagent 2 [50wt% sucrose, 25wt% urea, 10wt% 2,2,2′-nitrilotriethanol and 0.1% (v/v) Triton X-100] for 2 days^52^. The stained explants were imaged on a Zeiss Light-sheet Z.1 light-sheet microscope. The imaging was performed with a 5 × /0.16 detection lens and two light-sheet illuminations (left and right) using 5 × /0.1 illumination lenses. For each illumination (left and right) a separate image was captured for each channel imaged. The fluorescent signal was collected using a 460–500 nm emission filter/405 nm excitation laser (Hoechst), 660/LP nm emission filter/638 nm excitation laser (AlexaFluor-647 F-actin), 575–615 nm emission filter/561 nm excitation laser (Cy3-αSMA) and 505–545 emission filter/488 nm excitation laser (AlexaFluor-488 cytokeratin). Α Z stack of ∼1000 frames at 30 ms exposure on two CMOS (PCOEdge) cameras with 1920 × 1920 pixels images (2.329 μm pixel size and 4 μm optical sectioning steps) and lightsheet thickness ~ 10 μm was used. Zen software (Carl Zeiss; Germany) was used to combine the left and right illumination images at each Z-plane. Further data processing, rendering of Z-stacks and visualisation in 3D was performed using Imaris software (Andor Technology; Switzerland).

## Supplementary Information

Supplementary Information.
