# Supplementary material for: Ex vivo culture of intact human patient derived pancreatic tumour tissue
Source: Sci Rep. 2021 Jan 21;11:1944. doi: 10.1038/s41598-021-81299-0 (PMC7820421; doi:10.1038/s41598-021-81299-0)
Supplement: Supplementary file 1 — Supplementary Information. [file 41598_2021_81299_MOESM1_ESM.pdf]

Ex vivo culture of intact human patient derived pancreatic tumour tissue.

John Kokkinos[1,2], George Sharbeen[1], Koroush S. Haghighi[3], Rosa Mistica C. Ignacio[1], Chantal Kopecky[1], Estrella Gonzales-Aloy[1], Janet Youkhana[1], Paul Timpson[4,5], Brooke A. Pereira[4,5], Shona Ritchie[4,5], Elvis Pandzic[6], Cyrille Boyer[7,8], Thomas P. Davis[9,10], Lisa M. Butler[11,12], David Goldstein[1,3], Joshua A. McCarroll[2,13,14], Phoebe A. Phillips[1,2*].

[1]Pancreatic Cancer Translational Research Group, School of Medical Sciences, Lowy Cancer Research Centre, UNSW Sydney, NSW, Australia; [2]Australian Centre for Nanomedicine, ARC Centre of Excellence in Convergent Bio-Nano Science and Technology, UNSW Sydney, NSW, Australia; [3]Prince of Wales Hospital, Prince of Wales Clinical School, UNSW Sydney, NSW, Australia; [4]Cancer Theme, The Kinghorn Cancer Centre, Garvan Institute of Medical Research, NSW, Australia ; [5]St. Vincent's Clinical School, Faculty of Medicine, UNSW Sydney, NSW, Australia; [6]Biomedical Imaging Facility, Mark Wainwright Analytical Centre, Lowy Cancer Research Centre, UNSW Sydney, NSW, Australia; [7]Australian Centre for Nanomedicine, UNSW Sydney, NSW, Australia; [8]Centre for Advanced Macromolecular Design, School of Chemical Engineering, UNSW Sydney, NSW, Australia; [9]ARC Centre of Excellence in Convergent Bio-Nano Science and Technology and Australian Institute for Bioengineering and Nanotechnology, The University of Queensland, Queensland, Australia; [10]ARC Centre of Excellence in Convergent Bio-Nano Science and Technology, Monash Institute of Pharmaceutical Sciences, Monash University, VIC, Australia; [11]Adelaide Medical School and Freemasons Foundation Centre for Men's Health, University of Adelaide, SA, Australia; [12]South Australian Health and Medical Research Institute, Adelaide, SA, Australia; [13]Children's Cancer Institute, Lowy Cancer Research Centre, UNSW Sydney, NSW, Australia; [14]School of Women's and Children's Health, UNSW Sydney, NSW, Australia.

***Corresponding author**: Phoebe A. Phillips

Supplementary Figures

Patient ID	Age (yr)	Sex	Tumour type	Histological grade	Stage
Patient 1	77	Female	Pancreatic ductal adenocarcinoma	3	T2N2
Patient 2	86	Female	Pancreatic ductal adenocarcinoma	2	T2N2
Patient 3	67	Male	Pancreatic ductal adenocarcinoma	2	T3N2
Patient 4	71	Male	Pancreatic ductal adenocarcinoma	2	T1M0
Patient 5	70	Male	Pancreatic ductal adenocarcinoma	2	T2N0
Patient 6	74	Female	Pancreatic ductal adenocarcinoma	2	T2N1
Patient 7	64	Male	Pancreatic ductal adenocarcinoma	2	T3N2
Patient 8	74	Female	Pancreatic ductal adenocarcinoma	2	T3N0
Patient 9	50	Male	Pancreatic neuroendocrine tumour	1	T2N0
Patient 10	56	Female	Pancreatic neuroendocrine tumour	2	T3N1
Patient 11	28	Female	Pancreatic neuroendocrine tumour	1	T1N0
Patient 12	50	Female	Metastatic leiomyosarcoma to the pancreas	N/A	N/A
Patient 13	60	Female	Pancreatic ductal adenocarcinoma	2	T2N2
Patient 14	77	Female	Pancreatic ductal adenocarcinoma	2	T3N2
Patient 15	64	Male	Intrapancreatic cholangiocarcinoma	2	T3N1

Supplementary Table S1: Patient and tumour characteristics. Staging based on American Joint Committee on Cancer 8th Edition. Metastasis category not assessable by histology.

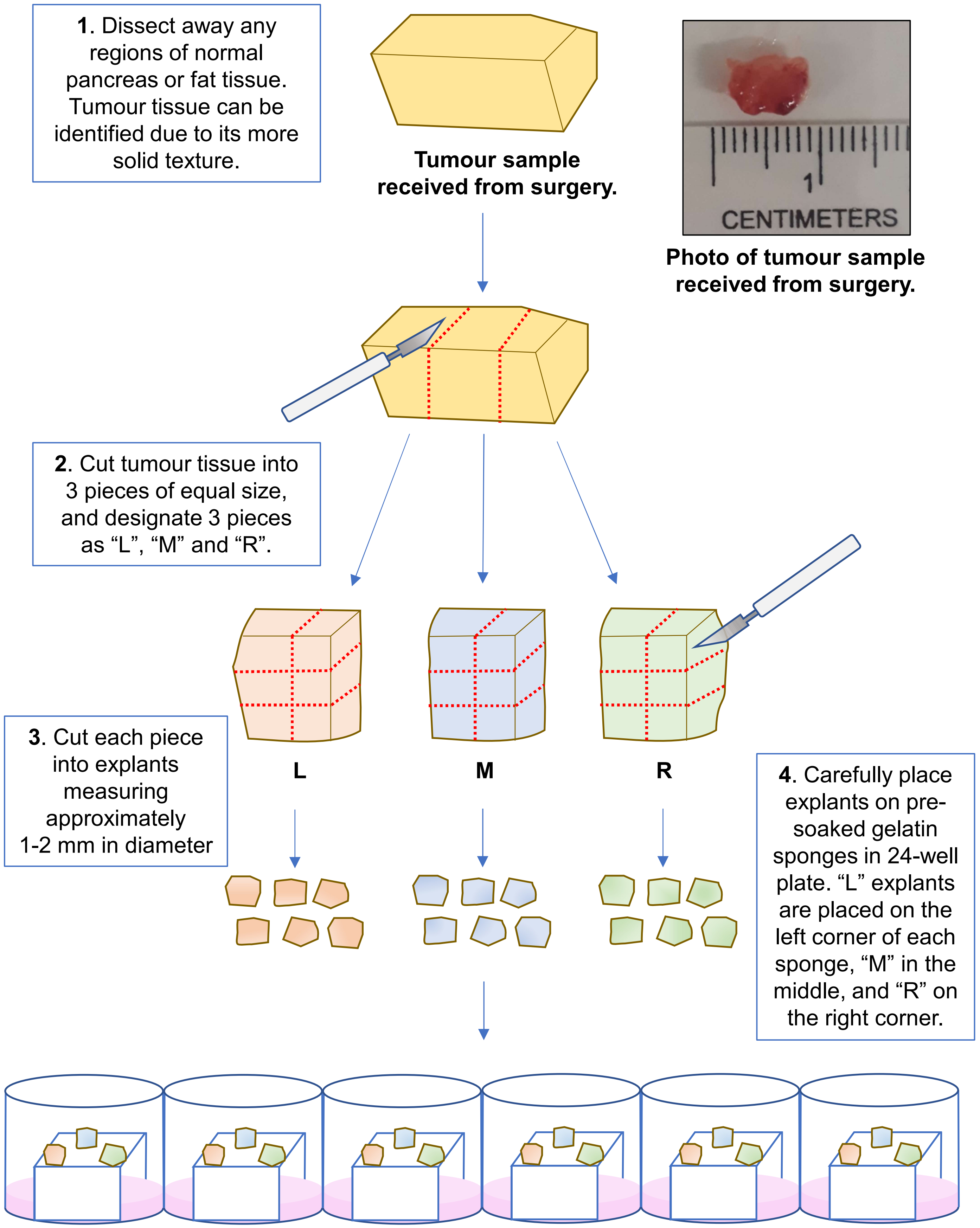

Supplementary Figure S1. Preparation of patient derived pancreatic ductal adenocarcinoma tumour explants from surgically resected tissue. Workflow diagram showing how tumour tissue is prepared and cut for explant culture.

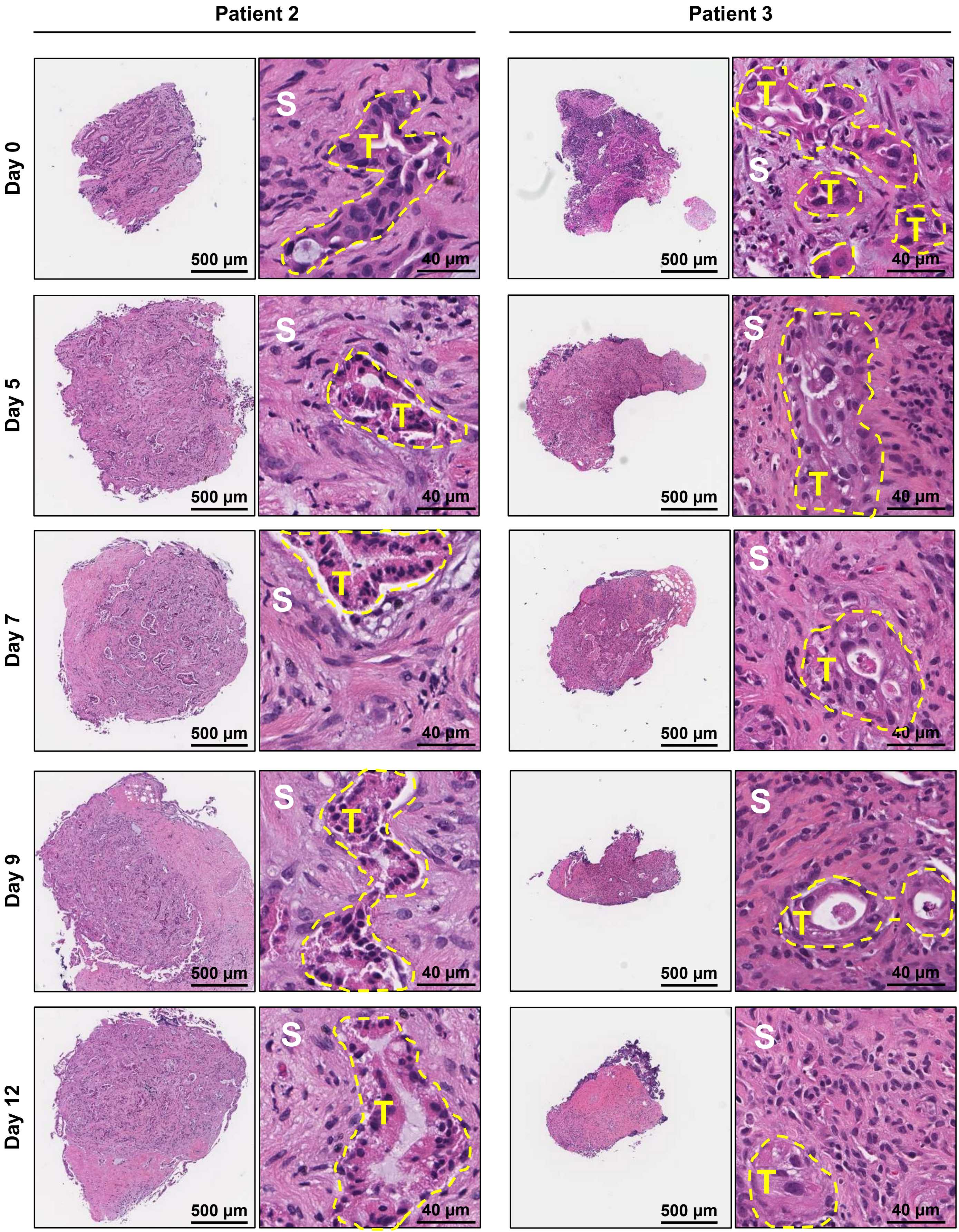

Supplementary Figure S2. H&E staining of human pancreatic ductal adenocarcinoma tumour explants from patients 2 and 3. Representative H&E images of patient 2 and 3 explants at low and high magnification from days 0-12. Tumour elements outlined in yellow and compartments labelled as tumour (T) and stroma (S).

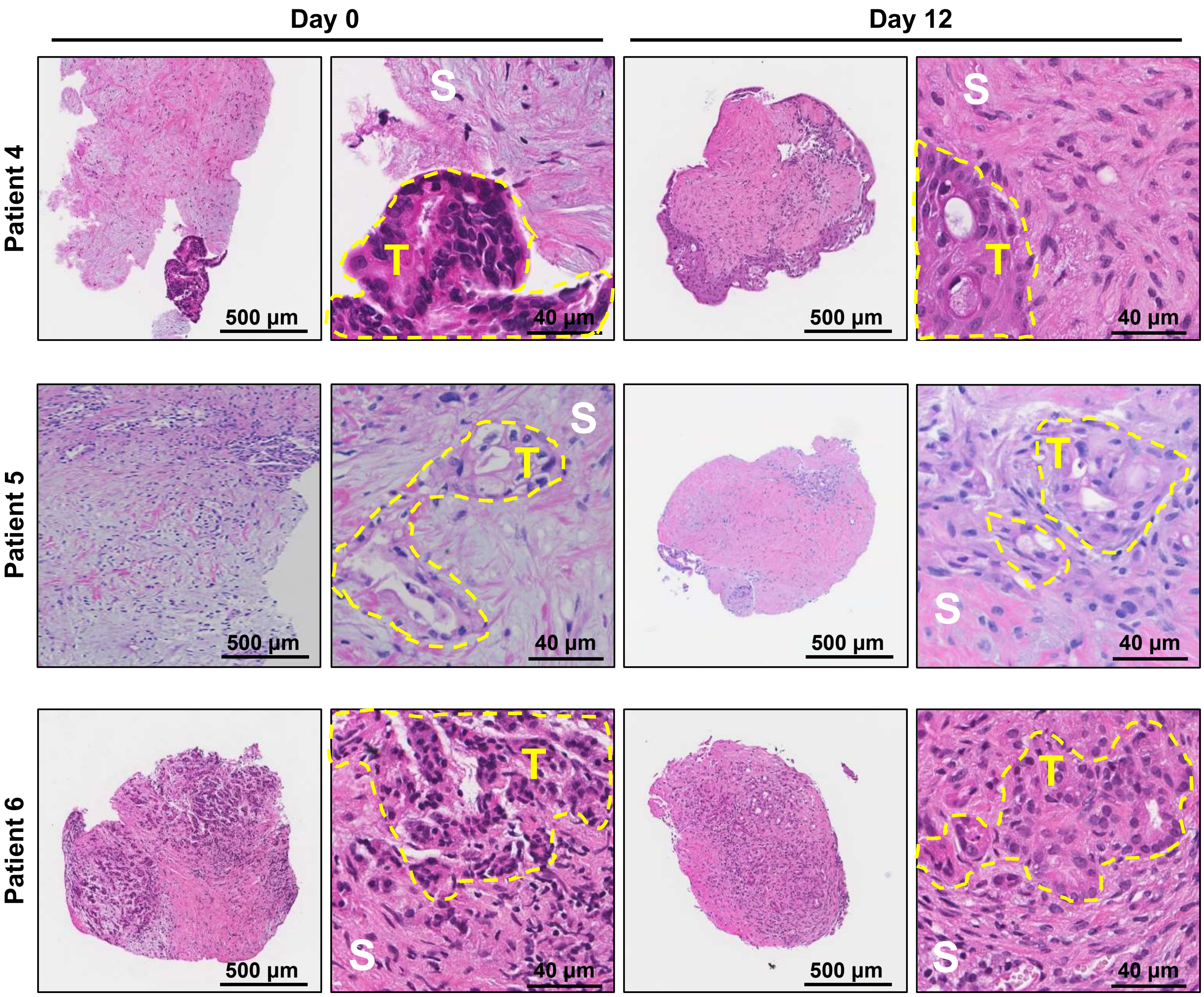

Supplementary Figure S3. H&E staining of human pancreatic ductal adenocarcinoma tumour explants from patients 4, 5 and 6. Representative H&E images of patient 4, 5 and 6 explants at low and high magnification from days 0 and 12. Tumour elements outlined in yellow and compartments labelled as tumour (T) and stroma (S).

Patient 2

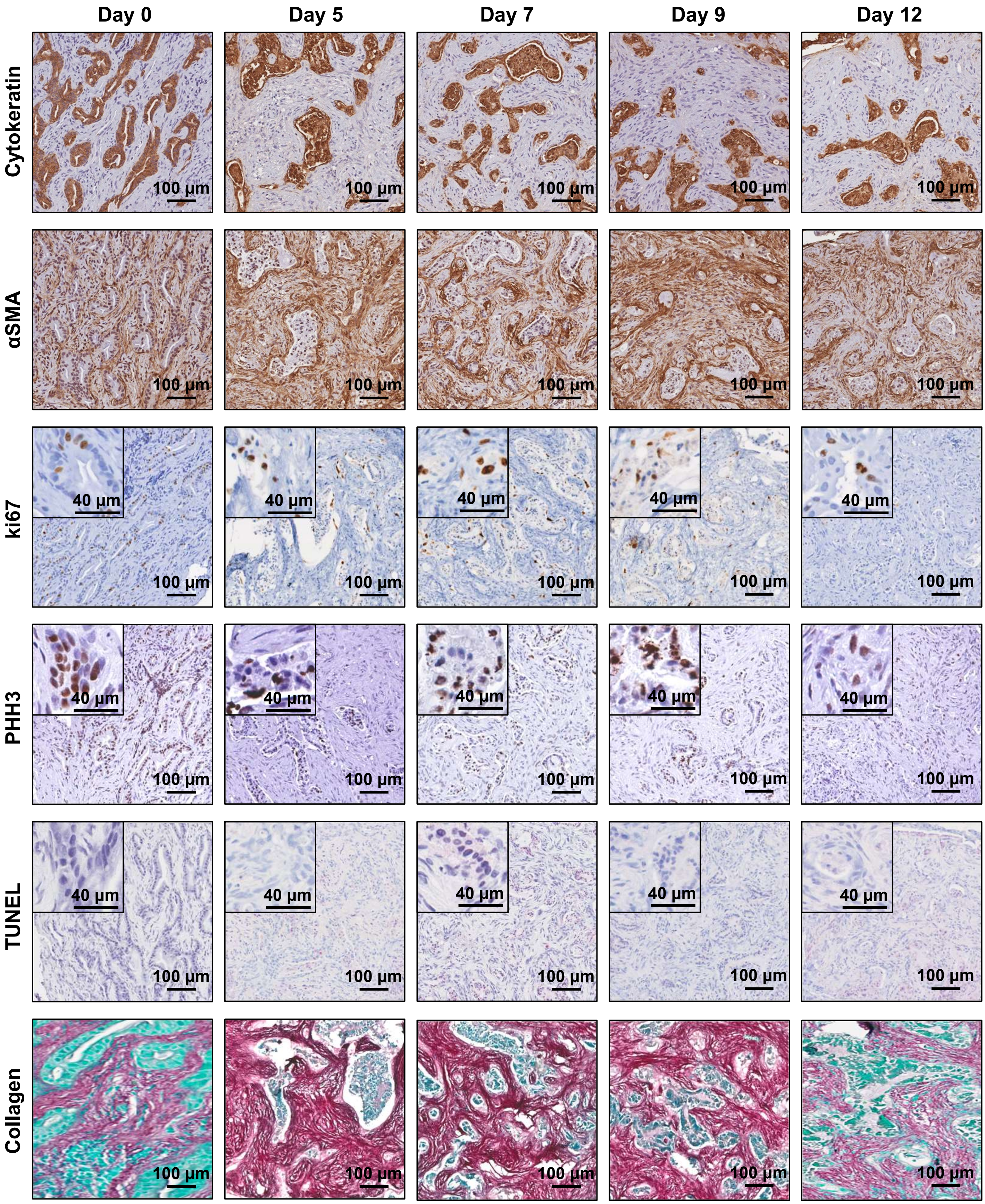

Supplementary Figure S4. Characterisation of patient 2 human pancreatic ductal adenocarcinoma explants from days 0-12. Immunohistochemistry was performed for cytokeratin, α-smooth muscle actin (αSMA), ki67, phospho-histone H3 (PHH3), TUNEL and collagen (picrosirius red/methyl green) on patient 2 PDAC explants from days 0-12. Insets for ki67, PHH3 and TUNEL stains show representative higher magnification views.

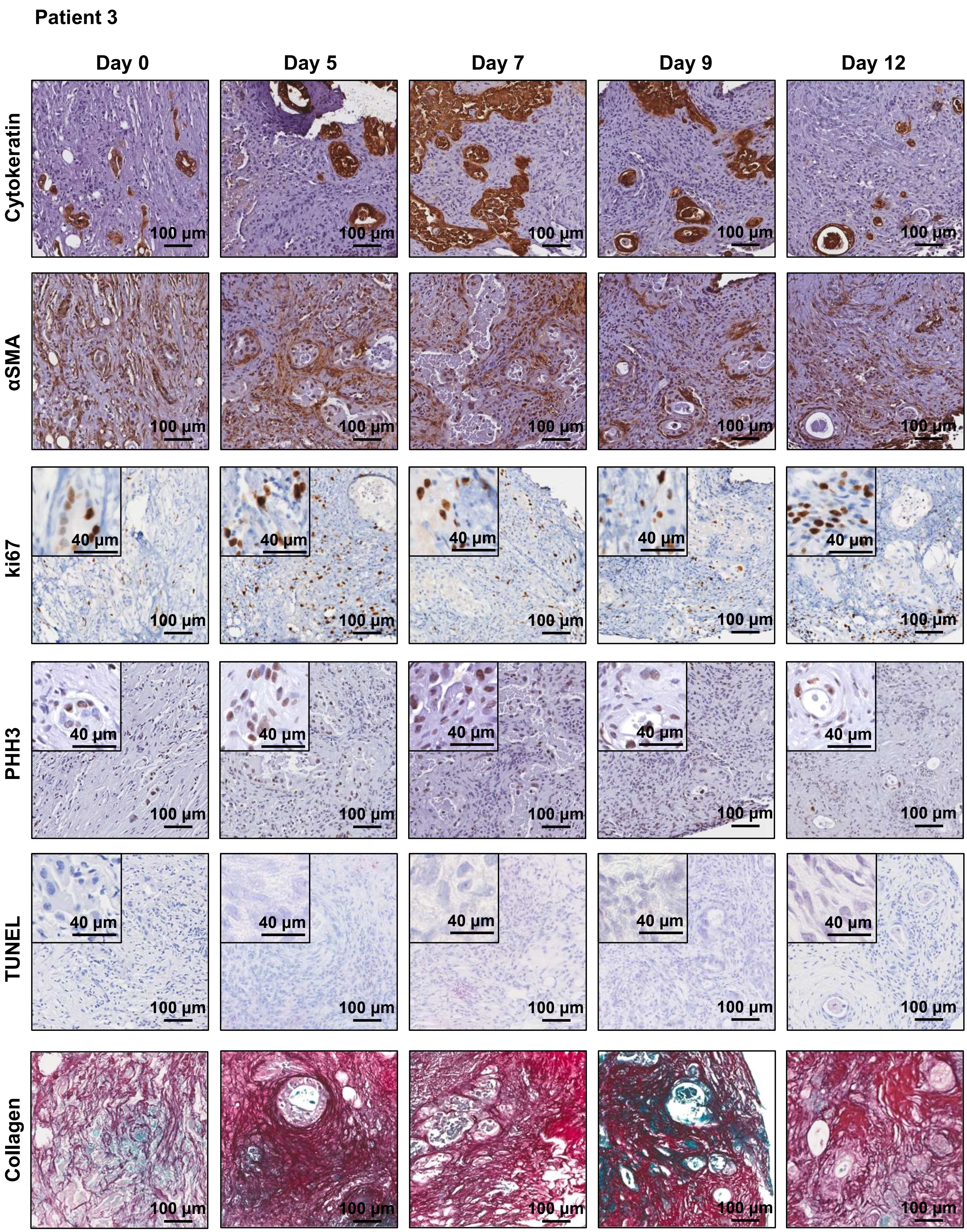

Supplementary Figure S5. Characterisation of patient 3 human pancreatic ductal adenocarcinoma explants from days 0-12. Immunohistochemistry was performed for cytokeratin, α-smooth muscle actin (αSMA), ki67, phospho-histone H3 (PHH3), TUNEL and collagen (picrosirius red/methyl green) on patient 3 PDAC explants from days 0-12. Insets for ki67, PHH3 and TUNEL stains show representative higher magnification views.

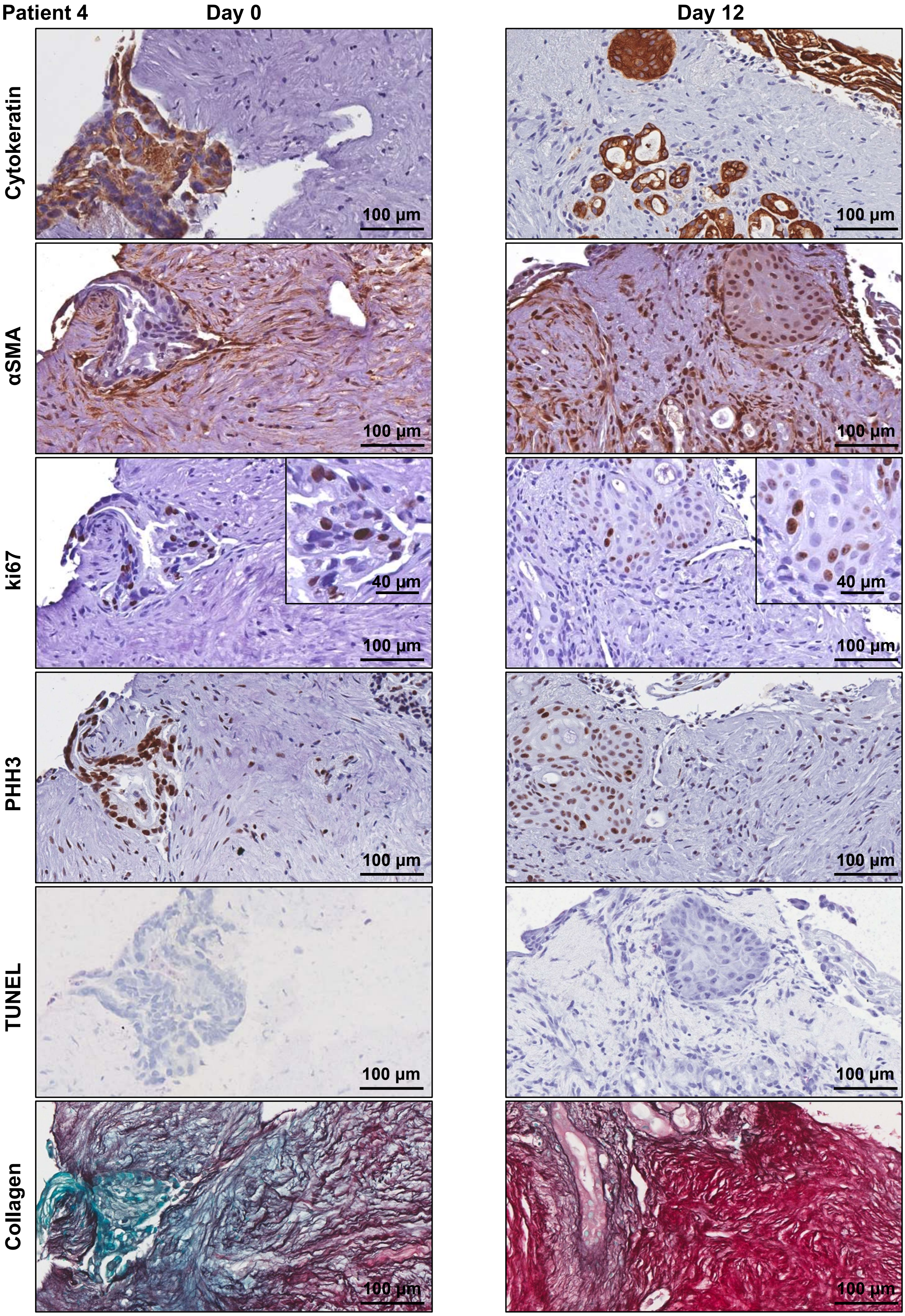

Supplementary Figure S6. Characterisation of patient 4 pancreatic ductal adenocarcinoma tumour explants at day 0 and day 12. Immunohistochemistry was performed for cytokeratin, α-smooth muscle actin (αSMA), ki67, phospho-histone H3 (PHH3), TUNEL and collagen (picrosirius red/methyl green) on patient 4 PDAC explants from day 0 and day 12. Insets for ki67 stains show representative higher magnification views.

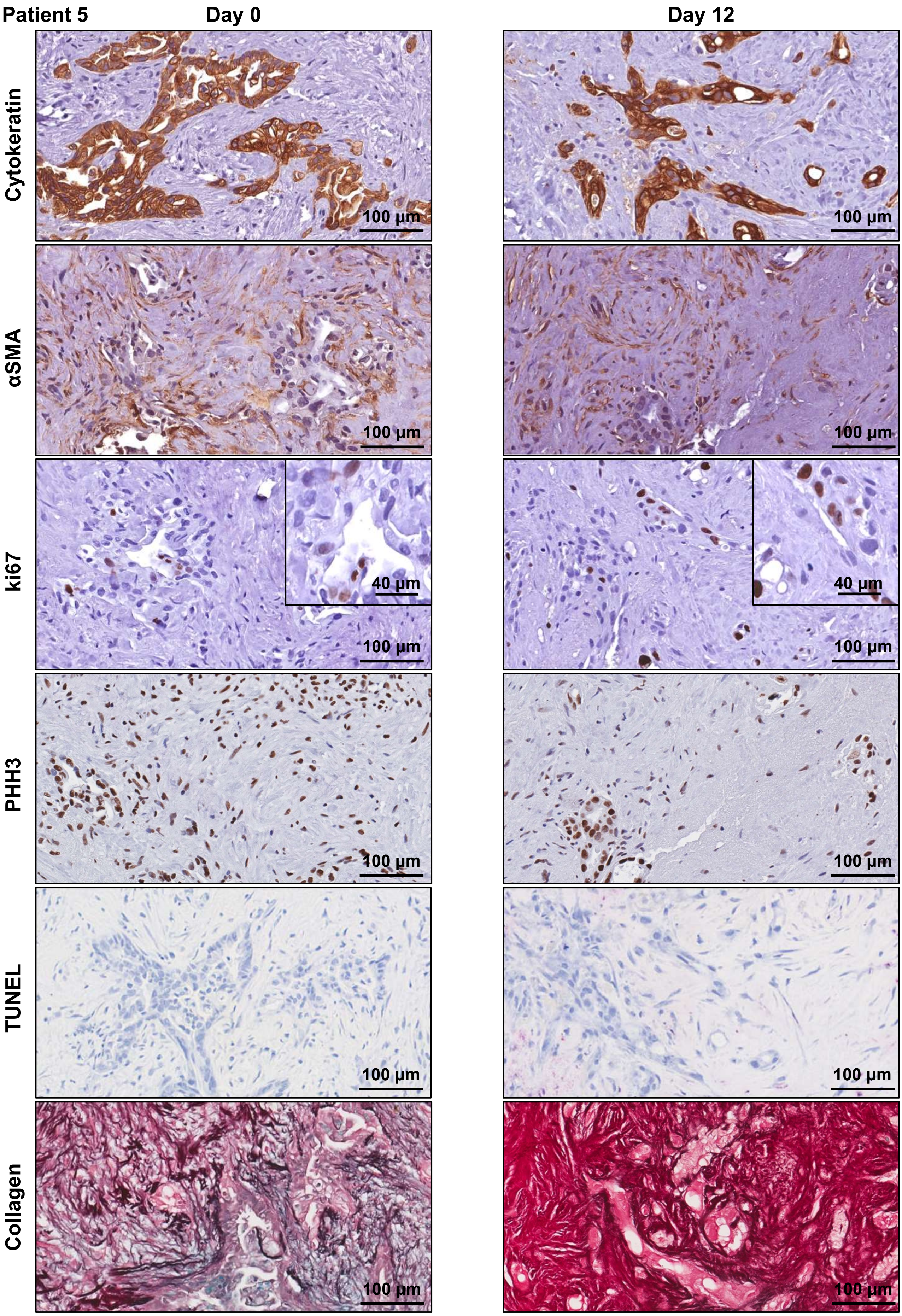

Supplementary Figure S7. Characterisation of patient 5 pancreatic ductal adenocarcinoma tumour explants at day 0 and day 12. Immunohistochemistry was performed for cytokeratin, α-smooth muscle actin (αSMA), ki67, phospho-histone H3 (PHH3), TUNEL and collagen (picrosirius red/methyl green) on patient 5 PDAC explants from day 0 and day 12. Insets for ki67 stains show representative higher magnification views.

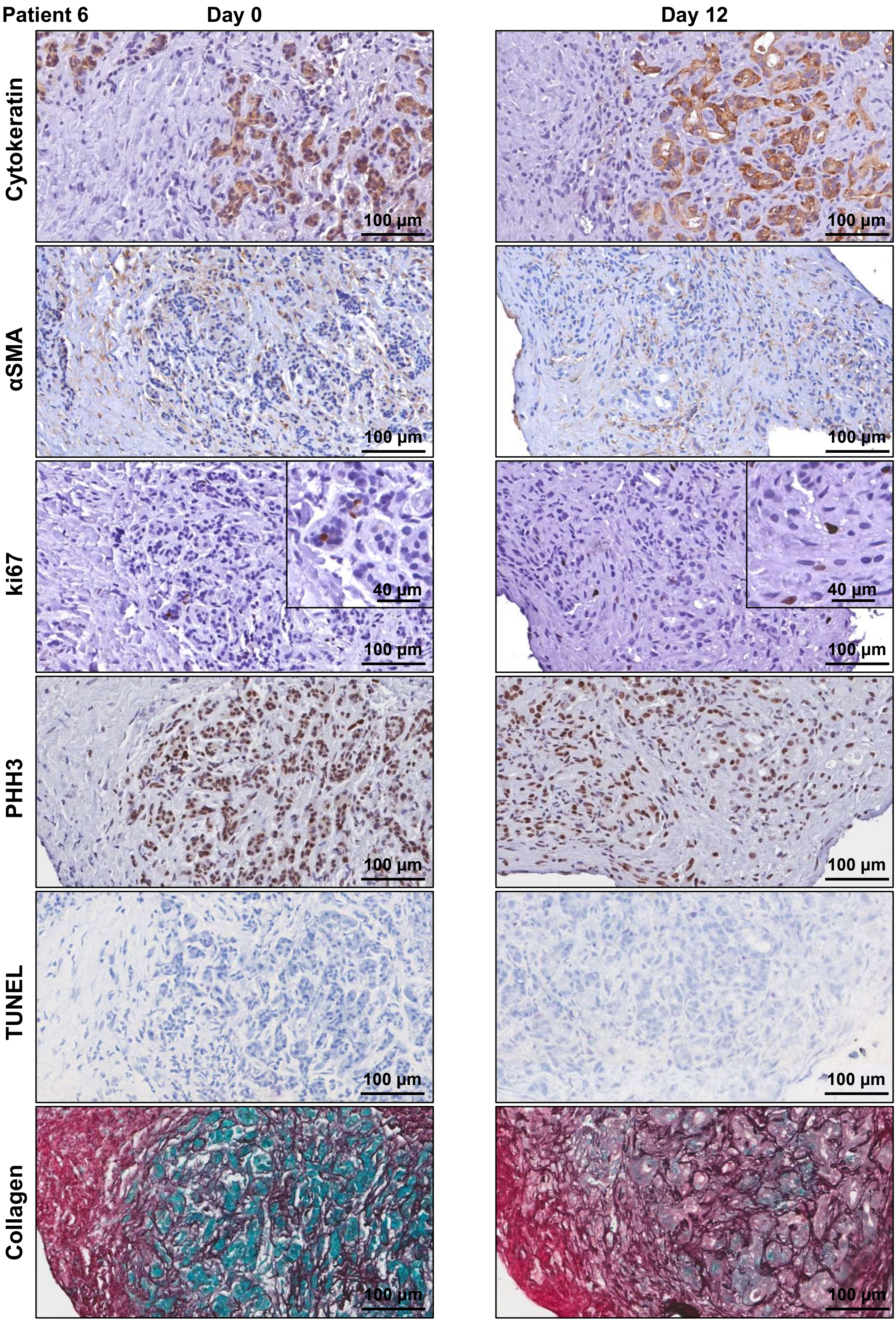

Supplementary Figure S8. Characterisation of patient 6 pancreatic ductal adenocarcinoma tumour explants at day 0 and day 12. Immunohistochemistry was performed for cytokeratin, α-smooth muscle actin (αSMA), ki67, phospho-histone H3 (PHH3), TUNEL and collagen (picrosirius red/methyl green) on patient 6 PDAC explants from day 0 and day 12. Insets for ki67 stains show representative higher magnification views.

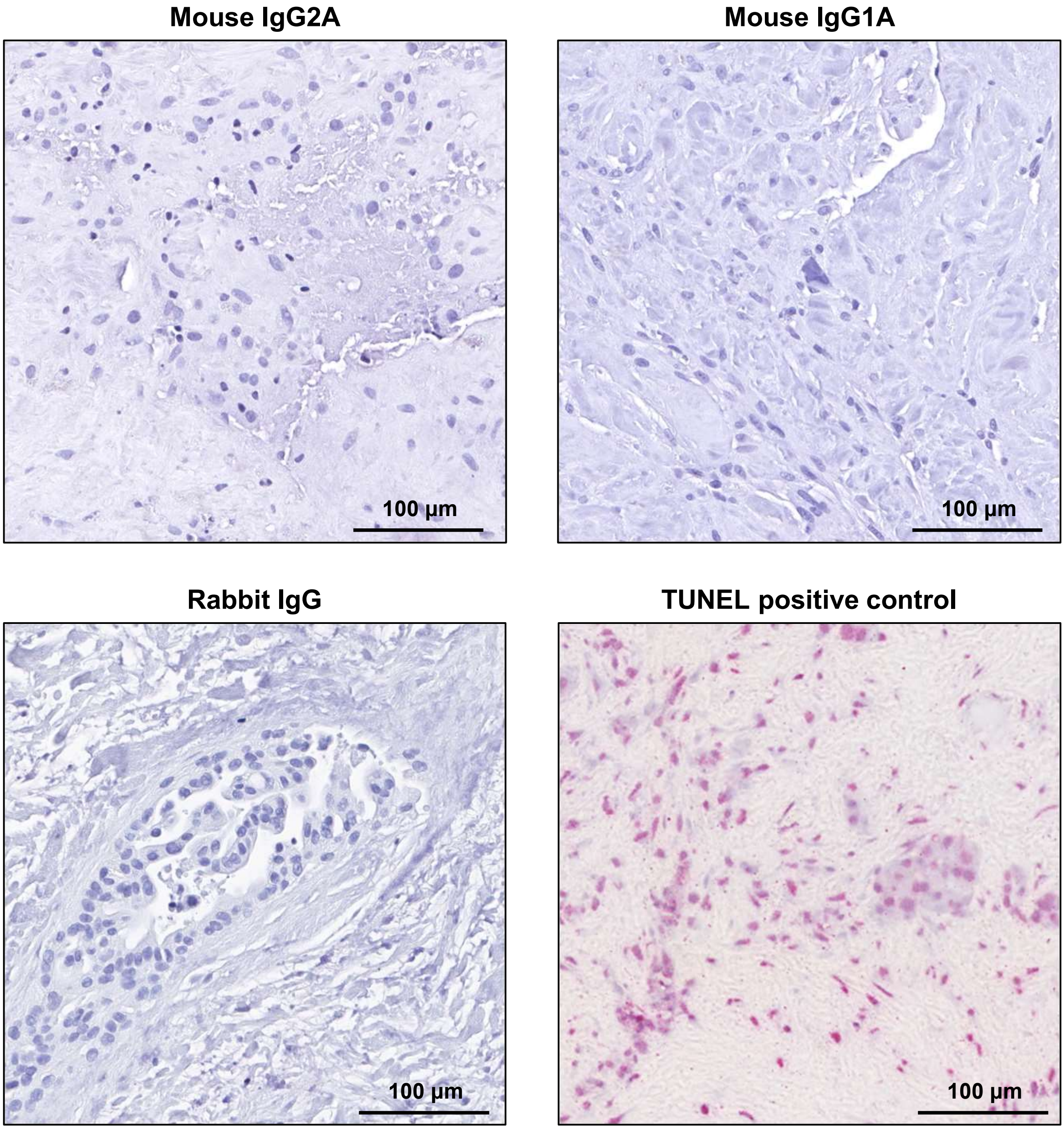

Supplementary Figure S9. Immunohistochemistry controls. Representative images showing each of the isotype control antibodies that were used for immunohistochemistry. Mouse IgG2A was used for α-Smooth Muscle Actin and p53 staining. Mouse IgG1A was used for cytokeratin, CD45, and bromodeoxyuridine staining. Rabbit IgG was used for ki67, synaptophysin and phospho-histone H3 staining. TUNEL positive control shows a human PDAC tissue section that was treated with 3 U/mL DNAse1 prior to proteinase K antigen retrieval.

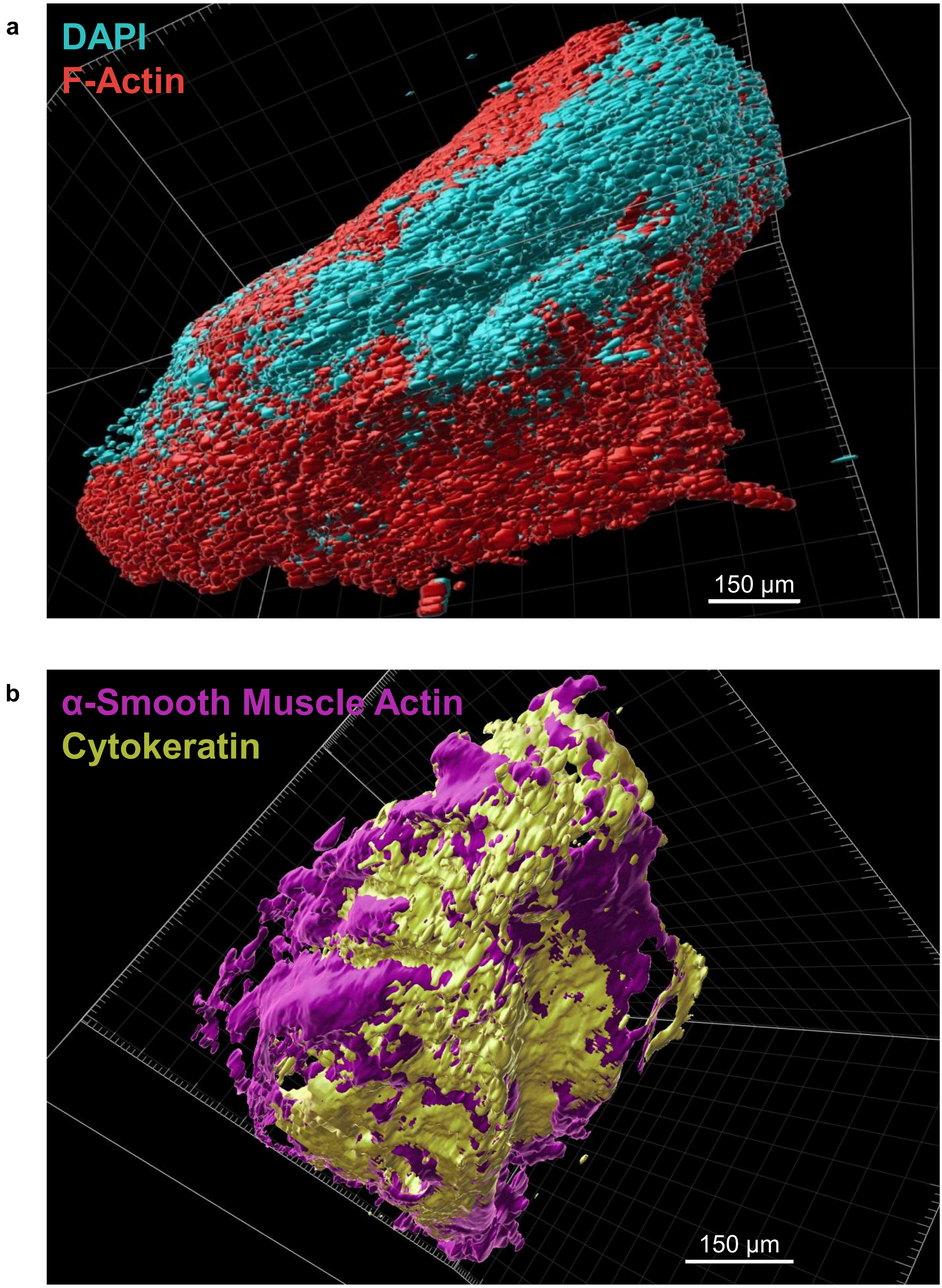

Supplementary Figure S10. 3D light-sheet microscopy imaging of human pancreatic ductal adenocarcinoma tumour explants. **(a-b)** 3D reconstructions of the whole-tissue explant showing **(a)** single cell nuclei (cyan) and F-actin (red) rich stromal cells and **(b)** cytokeratin-positive tumour elements (yellow) and α-smooth muscle actin-positive cancer associated fibroblasts (magenta).

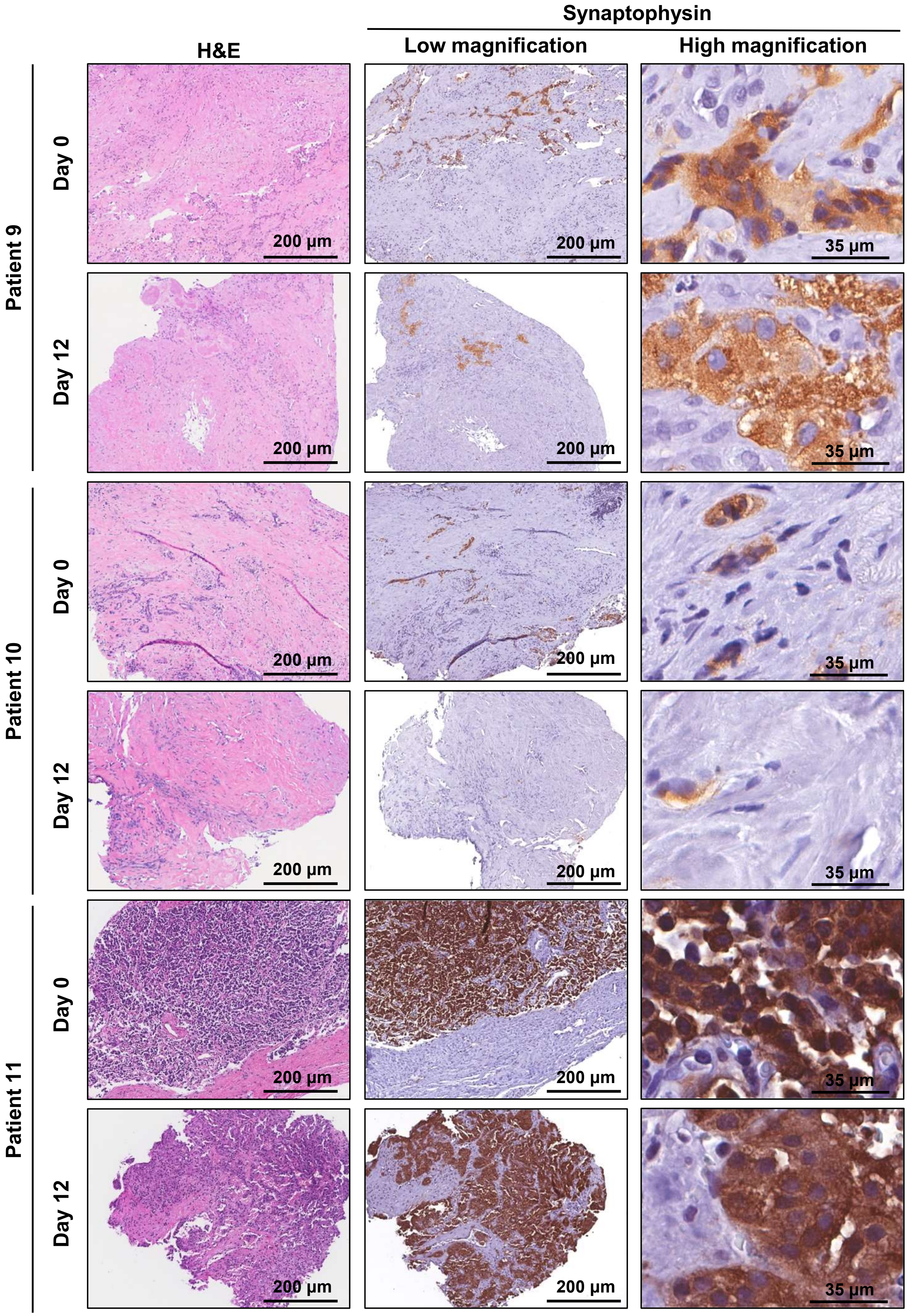

Supplementary Figure S11. Human explant culture of pancreatic neuroendocrine tumours from patients 9-11. Representative images of H&E staining of pancreatic neuroendocrine tumour explants at day 0 and day 12 of culture. Immunohistochemistry was performed for synaptophysin, a neuroendocrine marker, demonstrating that neuroendocrine tumour elements are maintained after 12 days of culture.

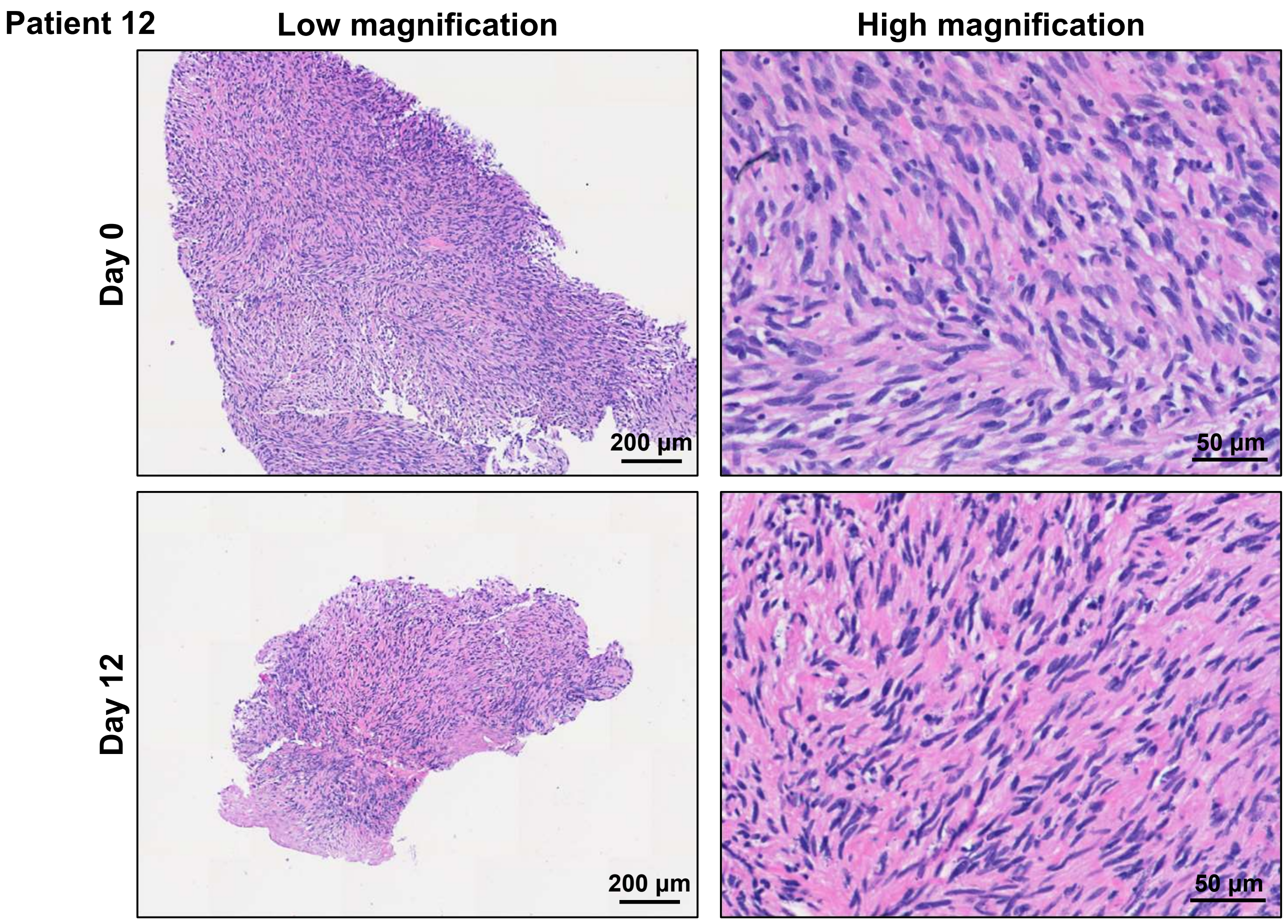

Supplementary Figure S12. Human explant culture of a rare metastatic leiomyosarcoma metastasis to the pancreas. Low and high magnification representative images of H&E staining of a metastatic leiomyosarcoma to the pancreas at day 0 and day 12 of culture.

Patient 7

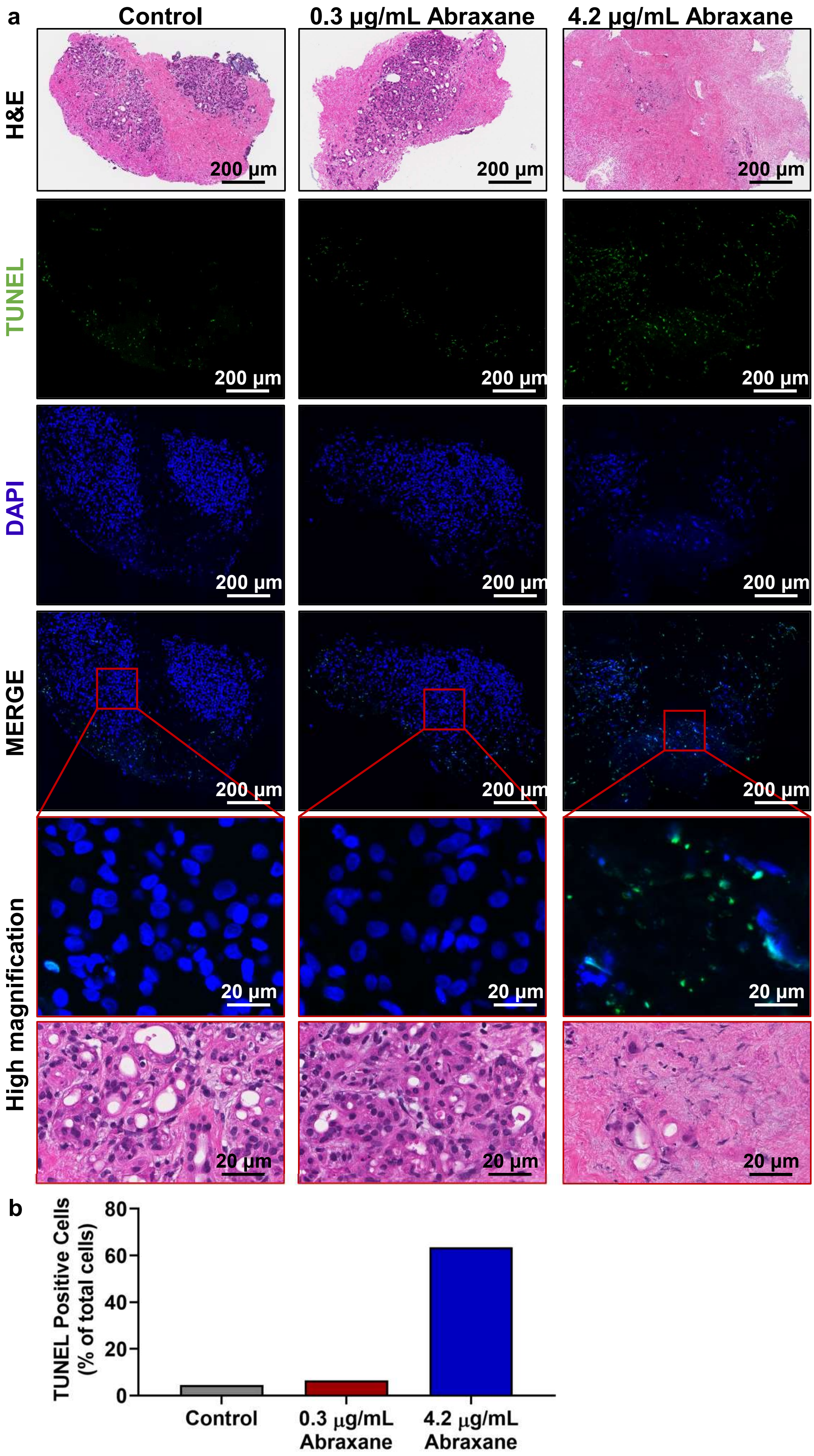

Supplementary Figure S13. Patient 7 pancreatic ductal adenocarcinoma tumour explants demonstrate response to Abraxane after 12 days. (a) Tumour explants from patient 11 were treated with or without 0.3 µg/mL or 4.2 µg/mL Abraxane on days 0, 3, 6, and 9, and then fixed on day 12. TUNEL staining was performed to assess levels of cell death. **(b)** Quantification of TUNEL positive cells using QuPath demonstrated high levels of cell death in 4.2 µg/mL Abraxane treated explants. Quantification was performed on a single explant per treatment.

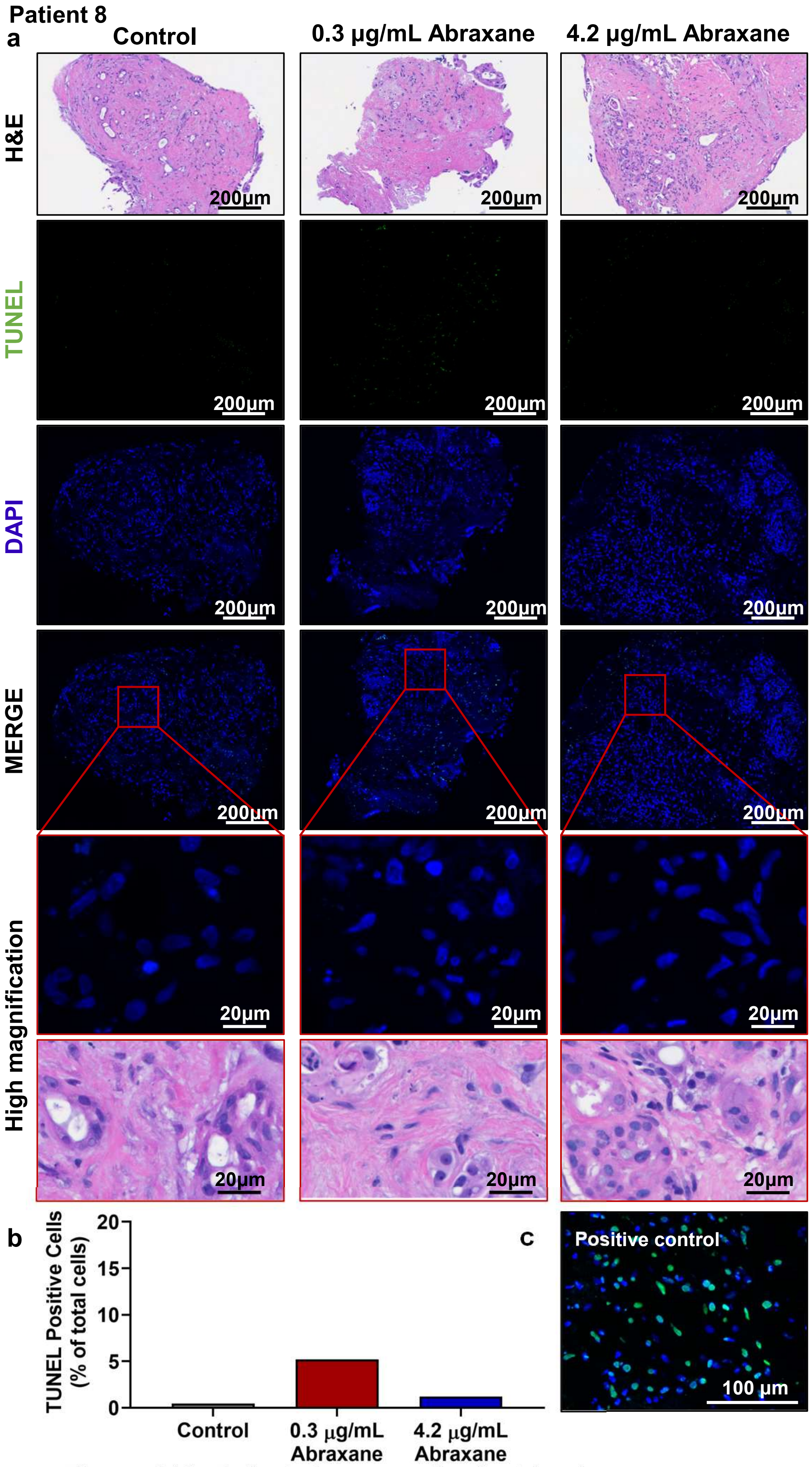

Supplementary Figure S14. Patient 8 pancreatic ductal adenocarcinoma tumour explants demonstrate lack of response to Abraxane after 12 days. (a) Tumour explants from patient 12 were treated with or without 0.3 µg/mL or 4.2 µg/mL Abraxane on days 0, 3, 6, and 9, and then fixed on day 12. TUNEL staining was performed to assess levels of cell death. **(b)** Quantification of TUNEL positive cells using QuPath demonstrated low levels of cell death in both doses of Abraxane treatment. Quantification was performed on a single explant per treatment. **(c)** TUNEL positive control of a human PDAC tissue section that was treated with DNAse1 prior to antigen retrieval.

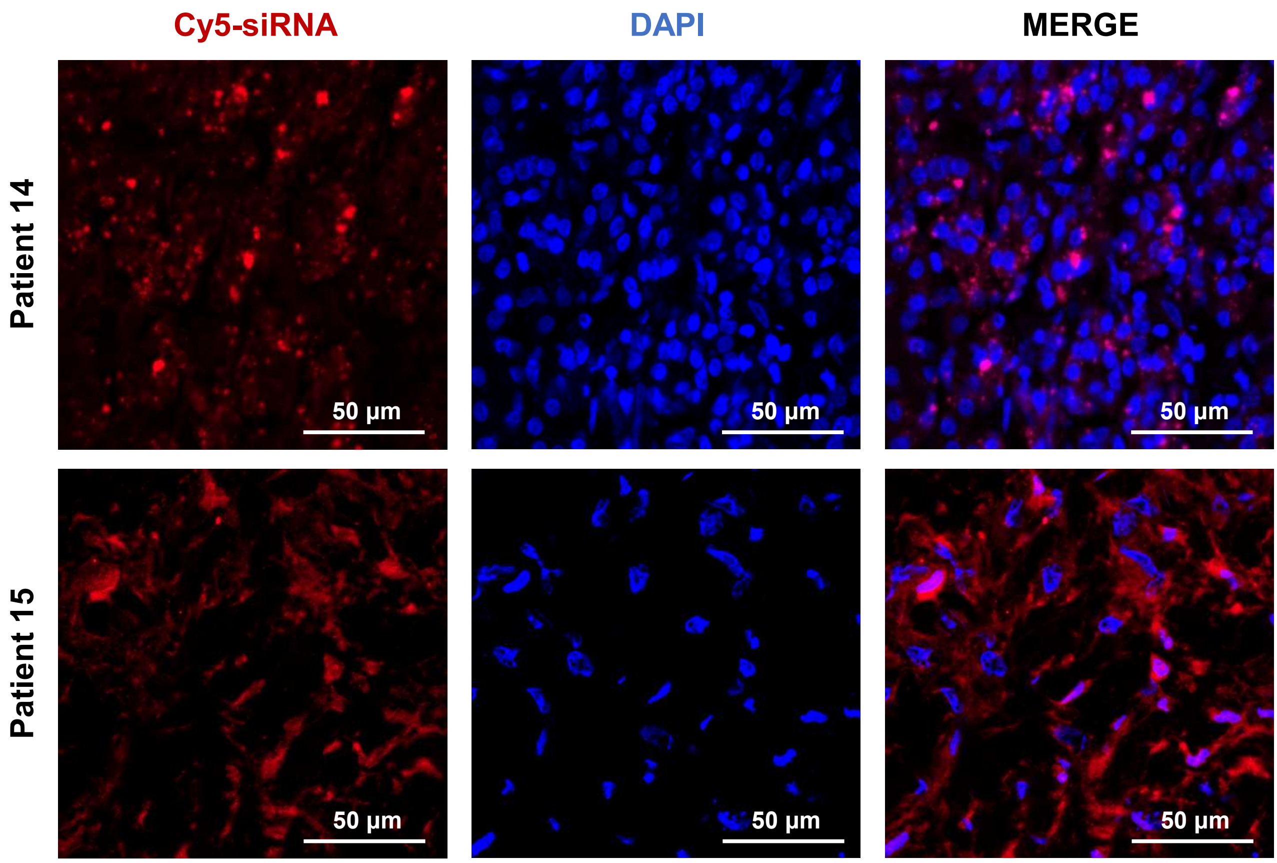

Supplementary Figure S15. Biodistribution of Star 3 nanoparticles in human explants from patients 14 and 15. Patient-derived explants were treated for 24 hours with or without Star 3 polymeric nanoparticles coupled to Cy5-siRNA. Representative images show biodistribution uptake of Cy5-siRNA throughout the entire explants. Patient 14 explants were taken from a patient with a pancreatic ductal adenocarcinoma, and patient 15 explants were taken from a patient with an intrapancreatic cholangiocarcinoma.
